# Cardioprotective glucose-lowering drugs, statins, and secondary major adverse cardiovascular events: a nationwide cohort study of individuals with type 2 diabetes and cardiovascular disease

**DOI:** 10.1186/s12933-026-03242-9

**Published:** 2026-06-16

**Authors:** Tummas Ternhamar, Mia Ø. Johansen, Børge G. Nordestgaard, Shoaib Afzal

**Affiliations:** 1 Department of Clinical Biochemistry, Copenhagen University Hospital - Herlev and Gentofte, Borgmester Ib Juuls Vej 73, Elevator 7, 4th Floor, N5, DK-2730 Herlev, Denmark; 2 The Copenhagen General Population Study, Copenhagen University Hospital - Herlev and Gentofte, Borgmester Ib Juuls Vej 73, 2730 Herlev, Denmark; 3 Department of Clinical Medicine, Faculty of Health and Medical Sciences, University of Copenhagen, Blegdamsvej 3, 2200 Copenhagen, Denmark; 4 Department of Clinical Biochemistry, Copenhagen University Hospital - Rigshospitalet, Blegdamsvej 9, 2100 Copenhagen, Denmark

**Keywords:** Diabetes, Sodium-glucose cotransporter-2 inhibitors, Glucagon-like peptide-1 receptor agonists, Statins, Cardiovascular disease

## Abstract

**Background:**

In type 2 diabetes, cardioprotective glucose-lowering drugs, including sodium-glucose cotransporter-2 inhibitors and glucagon-like peptide-1 receptor agonists, and statins reduce the risk of secondary major adverse cardiovascular events. No trials examined the combination of these drugs as withholding statins in high-risk individuals would be unethical. We tested the hypothesis that cardioprotective glucose-lowering drug and statin combined is associated with lower risk of secondary major adverse cardiovascular events than either drug alone.

**Methods:**

Individuals with type 2 diabetes and established cardiovascular disease from December 2012 through 2021 were identified via national Danish health registers. They were analyzed in an active comparator cohort including 15,404 individuals followed from treatment intensification with cardioprotective glucose-lowering drug or dipeptidyl peptidase-4 inhibitor and additionally in a time-varying cohort including 76,853 individuals with yearly updated treatment and covariate status. The treatment groups were: (i) no cardioprotective drug (no use of cardioprotective glucose-lowering drug or statin), (ii) cardioprotective glucose-lowering drug, (iii) statin, and (iv) cardioprotective glucose-lowering drug and statin. The primary outcome was a new major adverse cardiovascular event (myocardial infarction, stroke, or cardiovascular death).

**Results:**

During mean 2.7 and 4.7 years of follow-up, 1,843 and 23,051 had major adverse cardiovascular events in the active comparator and time-varying cohorts. In the active comparator cohort, when compared to nonusers of cardioprotective glucose-lowering drug or statin, multivariable adjusted hazard ratios of major adverse cardiovascular events were 0.82 (95% confidence interval: 0.67 to 1.02) for cardioprotective glucose-lowering drug, 0.85 (0.74 to 0.97) for statin, and 0.71 (0.60 to 0.84) for cardioprotective glucose-lowering drug and statin combined. Corresponding hazard ratios in the time-varying cohort were 0.77 (0.69 to 0.86), 0.73 (0.70 to 0.75), and 0.57 (0.54 to 0.60), respectively. When restricting the active comparator cohort to individuals entering observation between 2019 and 2021 with reduced statistical power, the corresponding hazard ratios were 0.86 (0.57 to 1.31), 0.89 (0.60 to 1.30), and 0.79 (0.54 to 1.14), respectively. In both cohorts *p* for interaction between cardioprotective glucose-lowering drug and statin was > 0.05.

**Conclusions and relevance:**

In individuals with type 2 diabetes and established cardiovascular disease, treatment with a cardioprotective glucose-lowering drug and statin in combination was associated with lower risk of secondary major adverse cardiovascular events than using either drug alone. This is important given the persistently suboptimal uptake of both drug classes in real-world practice. Some biases can never be completely excluded in real-world pharmacotherapy use studies such as this one, including confounding by indication, time-related or immortal time biases, and shifting standards of care, rendering causal interpretation unattainable; however, our results seemed consistent across numerous sensitivity analyses and designs.

**Graphical abstract:**

**Figure:**
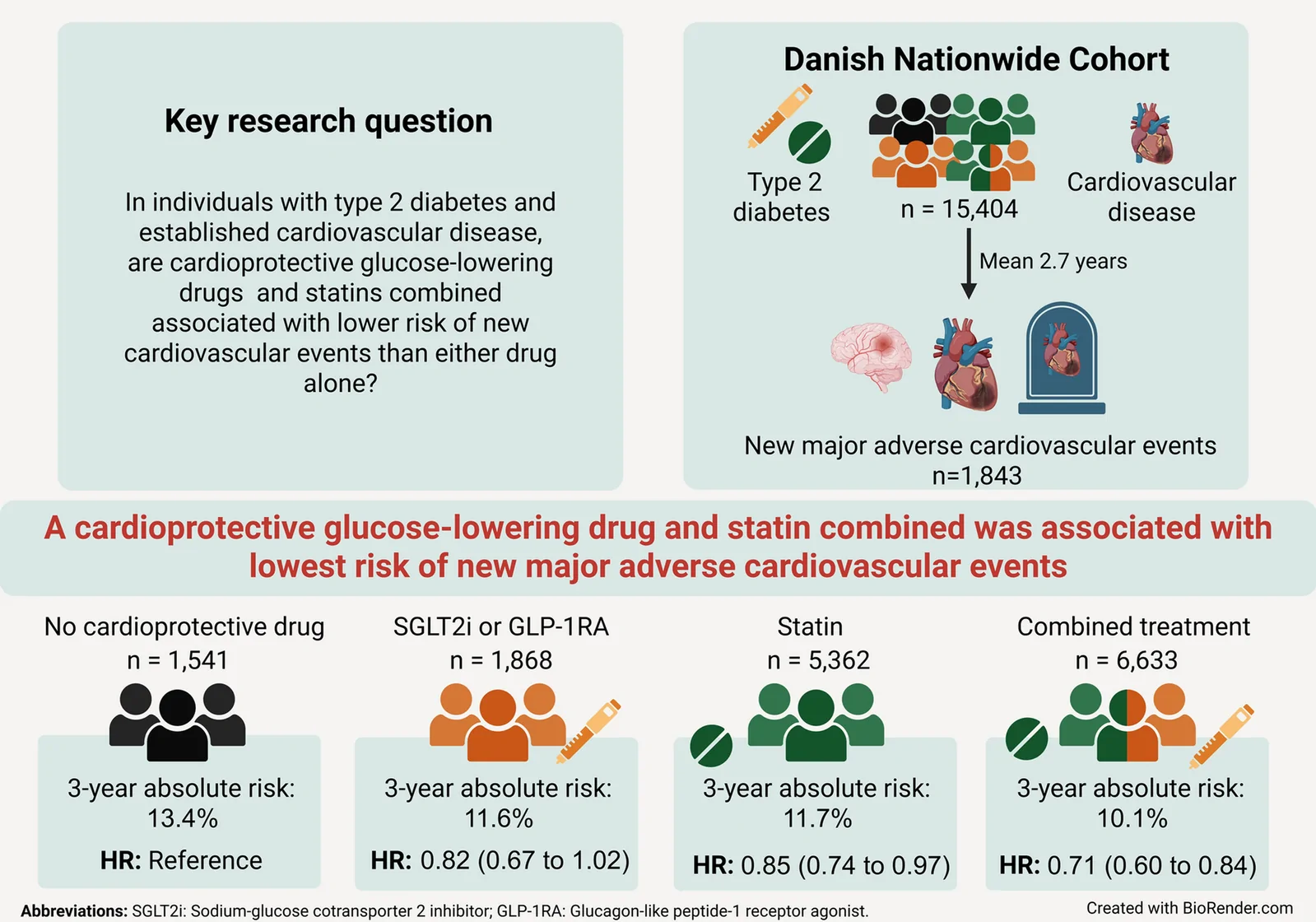

**Supplementary Information:**

The online version contains supplementary material available at 10.1186/s12933-026-03242-9.

## Research insights

**What is currently known on this topic?**

In diabetes, cardioprotective glucose-lowering drugs prevent secondary cardiovascular events. They include sodium-glucose cotransporter-2 inhibitors and glucagon-like peptide-1 receptor agonists. Statins likewise reduce the risk of secondary cardiovascular events in diabetes.

**What is the key research question?**

Are these drugs combined associated with lower risk of cardiovascular events than either alone?

**What is new?**

The question is explored in 76,853 individuals with diabetes and established cardiovascular disease. Combined treatment was associated with lowest risk of new cardiovascular events.

**How might this study influence clinical practice?**

Findings may reinforce use of both drug classes; they appear underused in real-world practice.

## Introduction

Statins are a cornerstone in the prevention of atherosclerotic cardiovascular disease in type 2 diabetes [1, 2]. Nevertheless, cardiovascular diseases remain the leading cause of morbidity, mortality, and health-care related spending associated with type 2 diabetes [3]. Novel cardioprotective glucose-lowering drugs, such as sodium-glucose cotransporter-2 inhibitors and glucagon-like peptide-1 receptor agonists, offer promising benefits beyond traditional strategies focused on managing glycemic control, hypertension, and dyslipidemia [1].

Several randomized clinical trials of sodium-glucose cotransporter-2 inhibitors and glucagon-like peptide-1 receptor agonists have demonstrated a reduction in major adverse cardiovascular events (MACE) [4–17]. These trials were designed to enroll high-risk individuals with type 2 diabetes, predominantly those with preexisting cardiovascular disease and baseline statin use [4–16]. As such, the efficacy of cardioprotective glucose-lowering drugs on secondary MACE has primarily been tested on a background of lipid-lowering treatment in high-risk patients with type 2 diabetes. Interactions or dependencies of using the two drug classes alone or in combination have never been tested in a trial. This question is important to address, as those with type 2 diabetes, even at high risk of MACE, remain inadequately treated with statins despite recommendations in international clinical guidelines [1, 2, 18–20]. We recently demonstrated in an unselected population of individuals with type 2 diabetes in Denmark, that combined use of cardioprotective glucose-lowering drugs and statins was associated with a lower risk of mortality compared to using either drug alone [21]. However, clinical guideline recommendations for combination therapy are strongest for secondary prevention of MACE and related mortality. Thus, it is important to determine benefits of combining cardioprotective glucose-lowering drugs and statins in secondary prevention of MACE and related mortality in real-world practice.

Using national Danish registers, we tested the hypothesis that using a cardioprotective glucose-lowering drug and statin in combination in type 2 diabetes is associated with lower risk of secondary MACE, including non-fatal stroke, myocardial infarction, or cardiovascular death, compared to using either drug class alone.

## Methods

### Data sources and study participants

Individual level data were obtained from national Danish registers from December 12, 2012, which with the introduction of dapagliflozin represents the earliest point when both sodium-glucose cotransporter-2 inhibitors and glucagon-like peptide-1 receptor agonists were available in Denmark [22], through December 31, 2021. The registers are linked through unique civil registration numbers assigned to individuals in Denmark at birth or immigration and used during administrative interactions with the health care system [23]. The health care system is publicly financed through taxes and provides comprehensive coverage for all residents in Denmark with essentially no loss to follow-up; individuals who die or emigrate are censored in the statistical analyses at the date of death or emigration.

Pharmacotherapy use was from the national Danish Registry of Medicinal Products Statistics [24]. Information on medical history was from the national Danish Patient Register holding World Health Organization International Classification of Diseases (ICD) system coded diagnoses and procedure codes [25]. Income and education levels were accessed through the Danish Income Statistics Register and the Danish Student Register [26, 27]. Information on death and emigration was from the national Danish Central Person Register, while the national Danish Register of Causes of Death provided information on primary, secondary, and tertiary causes of death identified by ICD codes [28].

Data on hemoglobin A1c, estimated glomerular filtration rate, and low-density lipoprotein cholesterol were from the national Danish laboratory registers [29]. These cover analyses from Danish hospital laboratories identified by Nomenclature, Properties, and Units (NPU) codes. Data became available at different timepoints in different Danish regions, with all regions covered from 2015 [29].

### Study design

First, in an active comparator cohort we contrasted new use of a cardioprotective glucose-lowering drug to a cardioneutral dipeptidyl peptidase-4 inhibitor [17], initiated as second-line glucose-lowering treatment after metformin in individuals with type 2 diabetes and established cardiovascular disease (history of myocardial infarction, stroke, atherosclerotic peripheral arterial disease, coronary revascularization, or heart failure, Supplementary Figure S1). Individuals were eligible if they had dispensed at least one prescription for metformin but no other glucose-lowering drug. Date of initial prescription for a cardioprotective glucose-lowering drug, either sodium-glucose cotransporter-2 inhibitor or glucagon-like peptide-1 receptor agonist, or dipeptidyl peptidase-4 inhibitor was the start of follow-up (index date). By aligning similar stages of diabetes progression, this design reduces confounding by indication arising from differences in diabetes duration, severity, and comorbidity profile [30]. The treatment groups were: (i) dipeptidyl peptidase-4 inhibitor (reference), (ii) cardioprotective glucose-lowering drug, (iii) dipeptidyl peptidase-4 inhibitor and statin, and (iv) cardioprotective glucose-lowering drug and statin. Use of statin and concomitant pharmacotherapies on the index date was defined by a minimum of two prescriptions in the two preceding calendar years. Hemoglobin A1c, estimated glomerular filtration rate, and low-density lipoprotein cholesterol values were the most recent value before start of follow-up (within 1 year before index date).

Second, in a time-varying cohort from January 2013 through December 2021, we studied everyone with type 2 diabetes and established cardiovascular disease (Supplementary Figure S2). They were eligible when having dispensed minimum one prescription for a glucose-lowering drug, irrespective of type. Variables not thought to be time-dependent including age, sex, diabetes duration, ethnicity, year of entry into observation, and education level were unchanged from baseline. Status of the other covariates was updated at the start of each calendar year (January 1, 2014 to January 1, 2021. The choice of yearly updates was to reflect the yearly status visit that patients with type 2 diabetes at minimum should attend in primary care practice in Denmark [31]. The earliest point of cohort entry was January 1, 2013, for individuals who had received any glucose-lowering drug prior to that year. For individuals naïve to glucose-lowering therapy who initiated treatment between 2013 and 2021, the index date was defined as January 1 of the following calendar year. By incorporating evolving markers of disease severity and treatment changes over time, it enabled assessment of the generalizability and robustness of conclusions drawn from the active comparator cohort, and explored potential adherence biases, that are probable with follow-ups as long as ten years. One prescription dispensed in the preceding calendar year designated use of treatments and concomitant pharmacotherapies. The treatment groups were: (i) no cardioprotective drug (neither cardioprotective glucose-lowering drug nor statin), (ii) cardioprotective glucose-lowering drug, (iii) statin, and (iv) cardioprotective glucose-lowering drug and statin.

In both cohorts, diagnosis of type 1 diabetes (ICD-10 code E10), gestational diabetes (ICD-10 code O24), insulin before age 30, or any glucose-lowering drug before age 15 resulted in exclusion. Follow-up was until outcome, death, emigration, or end of follow-up on December 31, 2021, for MACE, and December 31, 2022, for all-cause mortality. Codes used to identify pharmacotherapies, medical history, and laboratory analytes are detailed in Supplementary Tables S1–S4.

### Outcomes

The main outcome was MACE (nonfatal stroke, myocardial infarction, or cardiovascular death, Supplementary Table S2). Secondary outcomes were all-cause mortality and each subcomponent of MACE. A minimum 30-day grace period had to elapse between repeated diagnoses of myocardial infarction or stroke to consider it a new event, to lower risk of misclassifying follow-up registrations of a single event. Importantly, only new primary diagnoses for hospitalization with myocardial infarction were used, effectively excluding any reassessment for the same event and any outpatient follow-up. The validity of cardiovascular diagnoses in Danish registers are high, with a positive predictive value for recurrent myocardial infarction previously reported at 88% with a grace period as low as 24 h [32].

### Socioeconomic covariates

By disposable income within the calendar year preceding the index date, individuals were categorized into low (lowest quartile), middle (interquartile), and high (highest quartile) income. Income was adjusted using the Organization for Economic Cooperation and Development (OECD)-modified equivalence scale [33]. Education was categorized by International Standard Classification of Education (ISCED) ranking prior to index date into basic education (ISCED 0–2), high school or vocational (ISCED 3–4), or higher education (ISCED ≥ 5) [34].

### Statistical analysis

First, in the main analysis, multivariable Cox proportional hazards models were used to separately evaluate the associations of sodium-glucose cotransporter-2 inhibitors and glucagon-like peptide-1 receptor agonists with the risk of MACE and all-cause mortality. Second, although it cannot be assumed that sodium-glucose cotransporter-2 inhibitors and glucagon-like peptide-1 receptor agonists provide the same effect size on risk of MACE, guidelines recommend both drug classes comparably for the prevention of atherosclerotic cardiovascular disease in type 2 diabetes [1]. Therefore, we also pooled them into a single category, which allowed for assessment of the exposure of interest with more statistical power: guideline-directed cardioprotective glucose-lowering treatment and statin combination in secondary prevention. To further assess the robustness of the primary Cox multivariable regressions, a weighted marginal structural approach using a Cox model with inverse probability of treatment weighting to balance covariates was used to test associations with secondary MACE and all-cause mortality in both cohorts (further details in the Supplementary Methods). A standardized mean difference < 0.1 was used as the threshold to indicate adequate balance. The models were adjusted for sex, age, descent (Danish or other/descendant of other including individuals recently immigrated from another country or children of these individuals), year of entry into observation, income level, education level, diabetes duration (grouped by < 1 year, 1–3 years, 3–5 years, 5–7 years, or ≥ 7 years elapsed since first dispensed glucose-lowering drug), use of other lipid-lowering drug, beta-blocker, calcium channel blocker, renin-angiotensin system inhibitor, thiazide or thiazide-like drug, loop diuretic, potassium-sparing diuretic, nitrate, vitamin K antagonist, direct oral anticoagulant, antiplatelet, beta-2-agonist inhalant, anticholinergic inhalant, glucocorticoid inhalant, antidepressant, non-steroid anti-inflammatory drug, and glucocorticoid with systemic action, and medical history of nephropathy, neuropathy, retinopathy, hypertension, chronic obstructive pulmonary disease, atrial fibrillation, and venous thromboembolism. In the time-varying cohort glucose-lowering drugs not included in the treatment definition including insulin, metformin, dipeptidyl peptidase-4 inhibitor, sulfonylurea, or other glucose-lowering drug were included for adjustments in the Cox models. In separate models, we also included an interaction term between cardioprotective glucose-lowering drug and statin. Year of entry into observation was categorized by: “2012–2015”: before trials reported cardioprotective effects for cardioprotective glucose-lowering drugs [6, 13]; “2016–2018”: before international guidelines were updated to favor them in individuals with established cardiovascular disease [35]; and “2019–2021”: after guidelines were published. Missing data for income was included in the “middle” category comprising 0.3% and 0.03% in the active comparator and time-varying cohorts. No registered educational level was kept as “Unknown”. Otherwise, data for the covariates included in the main models was complete as presented in the baseline tables. More detail on the rationale for including each covariate is presented in Supplementary Table S5.

To explore absolute risks, we calculated standardized cumulative incidence curves for MACE with death from noncardiovascular causes as a competing event in the active comparator cohort. The G-formula for direct standardization was implemented using cause-specific Cox models with baseline covariates listed in Table 1, producing “covariate-adjusted” incidence curves [36, 37]. Two and three-year risk differences for statin alone versus combination therapy and for cardioprotective glucose-lowering drug alone versus combination therapy were assessed, corresponding to the median follow-up time in the active comparator cohort and the average follow-up time in randomized trials for cardioprotective glucose-lowering drugs, respectively [4–16].

**Table 1:** Baseline characteristics for the active comparator cohort

	Dipeptidyl peptidase-4 inhibitor (N = 1541)		Cardioprotective glucose-lowering drug (N = 1868)	Dipeptidyl peptidase-4 inhibitor and statin (N = 5362)	Cardioprotective glucose-lowering drug and statin (N = 6633)	Total (N = 15,404)
Covariates						
*Sex*						
Women	550 (35.7%)	538 (28.8%)		1604 (29.9%)	1733 (26.1%)	4425 (29%)
Age, years	70.6 (13.0)	64.1 (12.2)		70.6 (10.3)	66.9 (9.86)	68.2 (10.9)
*Year of entry into observation*						
2012–2015	493 (32.0%)	101 (5.4%)		1796 (33.5%)	333 (5.0%)	2723 (18%)
2016–2018	626 (40.6%)	413 (22.1%)		2222 (41.4%)	1212 (18.3%)	4473 (29%)
2019–2021	422 (27.4%)	1354 (72.5%)		1344 (25.1%)	5088 (76.7%)	8208 (53%)
*Descent*						
Danish	1411 (91.6%)	1694 (90.7%)		4880 (91.0%)	5953 (89.7%)	13,938 (90%)
Other/Decendant of other	130 (8.4%)	174 (9.3%)		482 (9.0%)	680 (10.3%)	1466 (10%)
*Income*						
Low	506 (32.8%)	398 (21.3%)		1691 (31.5%)	1237 (18.6%)	3832 (25%)
Middle	749 (48.6%)	888 (47.5%)		2648 (49.4%)	3429 (51.7%)	7714 (50%)
High	286 (18.6%)	582 (31.2%)		1023 (19.1%)	1967 (29.7%)	3858 (25%)
*Education*						
Basic	630 (40.9%)	663 (35.5%)		2303 (43.0%)	2419 (36.5%)	6015 (39%)
High school or vocational	685 (44.5%)	895 (47.9%)		2357 (44.0%)	3244 (48.9%)	7181 (47%)
Higher	176 (11.4%)	272 (14.6%)		560 (10.4%)	836 (12.6%)	1844 (12%)
Unknown	50 (3.2%)	38 (2.0%)		142 (2.6%)	134 (2.0%)	364 (2%)
*Diabetes duration*						
< 1 year	575 (37.3%)	814 (43.6%)		901 (16.8%)	1266 (19.1%)	3556 (23%)
1–3 years	283 (18.4%)	316 (16.9%)		1071 (20.0%)	1417 (21.4%)	3087 (20%)
3–5 years	234 (15.2%)	204 (10.9%)		1108 (20.7%)	1176 (17.7%)	2722 (18%)
5–7 years	160 (10.4%)	168 (9.0%)		899 (16.8%)	939 (14.2%)	2166 (14%)
> 7 years	289 (18.8%)	366 (19.6%)		1383 (25.8%)	1835 (27.7%)	3873 (25%)
*Pharmacotherapy*						
Other lipid-lowering drugs	65 (4.2%)	83 (4.4%)		245 (4.6%)	560 (8.4%)	953 (6%)
Beta-blockers	652 (42.3%)	698 (37.4%)		3328 (62.1%)	4144 (62.5%)	8822 (57%)
Calcium channel blockers	376 (24.4%)	416 (22.3%)		2118 (39.5%)	2584 (39.0%)	5494 (36%)
Renin-angiotensin system inhibitors	848 (55.0%)	1002 (53.6%)		4041 (75.4%)	4980 (75.1%)	10,871 (71%)
Thiazides, thiazide-like drugs	397 (25.8%)	392 (21.0%)		2066 (38.5%)	2216 (33.4%)	5071 (33%)
Loop diuretics	514 (33.4%)	478 (25.6%)		1691 (31.5%)	1642 (24.8%)	4325 (28%)
Potassium-sparing diuretics	233 (15.1%)	290 (15.5%)		752 (14.0%)	1083 (16.3%)	2358 (15%)
Nitrates	100 (6.5%)	64 (3.4%)		664 (12.4%)	853 (12.9%)	1681 (11%)
Vitamin K antagonists	233 (15.1%)	143 (7.7%)		704 (13.1%)	536 (8.1%)	1616 (10%)
Direct oral anticoagulants	198 (12.8%)	305 (16.3%)		594 (11.1%)	930 (14.0%)	2027 (13%)
Antiplatelets	610 (39.6%)	580 (31.0%)		4154 (77.5%)	4966 (74.9%)	10,310 (67%)
Beta-2 agonist inhalants	242 (15.7%)	295 (15.8%)		867 (16.2%)	1054 (15.9%)	2458 (16%)
Anticholinergic inhalants	150 (9.7%)	131 (7.0%)		487 (9.1%)	577 (8.7%)	1345 (9%)
Glucocorticoid inhalants	177 (11.5%)	175 (9.4%)		606 (11.3%)	663 (10.0%)	1621 (11%)
Antidepressants	143 (9.3%)	103 (5.5%)		572 (10.7%)	631 (9.5%)	1449 (9%)
Non-steroid anti-inflammatory drugs	268 (17.4%)	296 (15.8%)		945 (17.6%)	941 (14.2%)	2450 (16%)
Glucocorticoids with systemic action	131 (8.5%)	128 (6.9%)		431 (8.0%)	399 (6.0%)	1089 (7%)
*Medical history*						
Nephropathy	301 (19.5%)	281 (15.0%)		818 15.3%)	996 (15.0%)	2396 (16%)
Neuropathy	152 (9.9%)	89 (4.8%)		606 (11.3%)	381 (5.7%)	1228 (8%)
Retinopathy	79 (5.1%)	52 (2.8%)		289 (5.4%)	284 (4.3%)	704 (5%)
Hypertension	871 (56.5%)	941 (50.4%)		3521 (65.7%)	4012 (60.5%)	9345 (61%)
Chronic obstructive pulmonary disease	267 (17.3%)	216 (11.6%)		848 (15.8%)	837 (12.6%)	2168 (14%)
Atrial fibrillation	501 (32.5%)	500 (26.8%)		1339 (25.0%)	1452 (21.9%)	3792 (25%)
Venous thromboembolism	87 (5.6%)	110 (5.9%)		286 (5.3%)	360 (5.4%)	843 (5%)
*Laboratory data*						
Hemoglobin A1c, mmol/mol	62 [54; 74]	61 [52; 77]		60 [53; 69]	58 [51; 67]	59 [52; 69]
LDL cholesterol, mmol/L	2.4 [1.7; 3.1]	2.3 [1.6; 3.1]		1.7 [1.3; 2.1]	1.6 [1.2; 2.0]	1.7 [1.3; 2.3]
Estimated GFR, mL/min per 1.73 m2	75 [48; 92]	85 [68; 98]		72 [47; 90]	83 [65; 94]	80 [58; 93]
Eligibility						
*Cardiovascular disease*	1,541 (100%)	1,868 (100%)		5,362 (100%)	6,633 (100%)	15,404 (100%)
Myocardial infarction	397 (25.8%)	581 (31.1%)		2039 (38.0%)	2781 (41.9%)	5798 (38%)
Stroke	467 (30.3%)	446 (23.9%)		1555 (29.0%)	1563 (23.6%)	4031 (26%)
Coronary revascularization	331 (21.5%)	599 (32.1%)		2551 (47.5%)	3618 (54.5%)	7099 (46%)
Peripheral arterial disease	245 (15.9%)	173 (9.3%)		900 (16.8%	958 (14.4%)	2276 (15%)
Heart failure	648 (42.1%)	838 (44.9%)		1684 (31.4%)	2128 (32.1%)	5298 (34%)

Baseline characteristics for the active comparator cohort. Continuous variables are reported as mean (standard deviation) or median [interquartile range], and categorical variables as count (percentage). LDL cholesterol in mg/dl is mmol/L multiplied by 38.67. Hemoglobin A1c in % is (mmol/mol multiplied by 0.095) + 2.15. *LDL*: low-density lipoprotein; *GFR*: glomerular filtration rate

Several subgroup and sensitivity analyses were conducted to ensure the results from the pooled cardioprotective glucose-lowering drug treatment groups were robust. Due to prohibitively long computation times when using marginal structural models with inverse-probability of treatment weights, all subgroup and sensitivity analyses were conducted using the multivariable-adjusted Cox models, since main analyses did not show major differences between the models. First, to assess whether the calendar year, and associated changes in standards of care, was a major confounder driving the findings, the Cox models were stratified by year of entry into observation (2019–2021, 2016–2018, and 2012–2015), each with reduced statistical power compared with the overall study design as is also the case for other stratified or restricted analyses described below. Second, Cox models for the main outcomes were stratified by sex assigned at birth (male or female). Third, to assess whether residual biases relating to different treatment durations were a concern, Cox models were stratified by statin exposure prior to the index date (time since first prescription: < 1 year, 1–3 years, 3–5 years, or > 5 years). Fourth, medication persistence and adherence, particularly for statins, vary substantially over time; therefore, a sensitivity analysis was conducted for the time-varying cohort where statin use was strictly defined by a proportion of days covered > 80% in the preceding year. Fifth, we included a sensitivity analysis using an expanded grace period of 90 days to classify new events to exclude any miscoding of a secondary MACE event during follow-up. Sixth, laboratory values were not included in the main models due to a substantial proportion of missing data (ranging from 4 to 34% across cohorts because we only have full coverage of all laboratory data in Denmark from 2015 onwards and possibly because not all included patients had hemoglobin A1c and low-density lipoprotein cholesterol measured), and because adjusting for low-density lipoprotein cholesterol would constitute adjusting for a mediator in some analyses. To explore whether the inclusion of these variables would alter the results, two sensitivity analyses were conducted: one adding hemoglobin A1c and estimated glomerular filtration rate to the Cox models, and another further adding low-density lipoprotein cholesterol. Seventh, to evaluate the outcomes of new stroke, myocardial infarction, or cardiovascular death in a population with more strictly defined cardiovascular disease, a sensitivity analysis restricted the cohort to individuals with a prior myocardial infarction. Eighth, to explore the addition of cardioprotective glucose-lowering drugs and statins in individuals already on optimal treatment, we assessed secondary MACE and all-cause mortality using Cox models restricted to individuals on antiplatelet and renin-angiotensin system inhibitor therapies at baseline. Ninth, to supplement the cause-specific hazard ratios estimated from the primary Cox models, multivariable adjusted Fine and Gray models treating noncardiovascular death as a competing risk were included for the active comparator cohort. Finally, if the standardized mean difference for any covariate remained > 0.1 after weighting in the models using inverse probability for treatment weighting, a further analysis was conducted that explicitly adjusted for these imbalanced variables in the weighted Cox outcome model.

Continuous variables are reported by mean and standard deviations (SD) or median and interquartile range and categorical variables by count and percentages. For Cox models the proportional hazards assumption was evaluated by plotting scaled Schoenfeld residuals by transformed time. These suggested no major violations. Confidence intervals for the clustered models including time-varying covariates were constructed using robust standard errors. Cox model results from stepwise adjustment: unadjusted; adjusted for age, sex, and ethnicity; additionally adjusted for general demographic variables (year of entry into observation, income level, education level, and diabetes duration); and fully adjusted including medical history and concomitant pharmacotherapy, are also reported. Analyses were performed using R version 4.4.1 (further details in the Supplementary Appendix, Supplementary Methods).

## Results

In the active comparator cohort, during a mean follow-up of 2.7 (SD 2.3) years, 41,289 years at risk in total, 1,843 secondary MACE events occurred, and during mean 3.6 (SD 2.4) years, 56,393 years at risk in total, 3,192 deaths occurred. In the time-varying cohort, during mean follow-up of 4.7 (SD 3.2), 365,042 years at risk in total, 23,051 secondary MACE events occurred, and during mean follow-up of 5.4 (SD 3.1), 411,853 years at risk in total, 34,347 deaths occurred.

About two thirds were men in the active comparator cohort, statin users were older, while higher income level was more frequent in individuals using cardioprotective glucose-lowering drugs (Table 1). Individuals initiating cardioprotective-glucose lowering drugs entered follow-up more often during the later years of the observation period. Duration of metformin treatment prior to baseline in the active comparator cohort was 4.6 years (SD 3.9), and 460 initiated metformin and a second-line glucose-lowering drug simultaneously at baseline. Characteristics were similar at baseline (time 0) in the time-varying cohort (Table 2), and use of cardioprotective glucose-lowering drugs increased with time (Supplementary Table S6). Compared to the active comparator cohort, individuals in the time-varying cohort had, on average, a one-year longer duration of diabetes and a lower estimated glomerular filtration rate (compare Tables 1 and 2). Conversely, baseline hemoglobin A1c was higher in the active comparator cohort, who entered follow-up when intensifying treatment with a second-line glucose-lowering drug.

**Table 2:** Baseline characteristics (time 0) for the time-varying cohort

	No cardioprotective drug (N = 13,268)		Cardioprotective glucose-lowering drug (N = 886)	Statin (N = 57,808)	Cardioprotective glucose-lowering drug and statin (N = 4891)	Total (N = 76,853)
Covariates						
*Sex*						
Women	5319 (40.1%)		330 (37.2%)	19,013 (32.9%)	1415 (28.9%)	26,077 (34%)
*Age, years*	72.9 (12.8)		62.5 (11.9)	70.5 (10.3)	64.5 (9.41)	70.4 (10.9)
Year of entry into observation						
2013–2015	9706 (73.2%)		425 (48.0%)	43,331 (75.0%)	3398 (69.5%)	56,860 (74%)
2016–2018	1918 (14.5%)		94 (10.6%)	7415 (12.8%)	301 (6.2%)	9728 (13%)
2019–2021	1644 (12.4%)		367 (41.4%)	7062 (12.2%)	1192 (24.4%)	10,265 (13%)
*Descent*						
Danish	12,272 (92.5%)		815 (92.0%)	52,816 (91.4%)	4486 (91.7%)	70,389 (92%)
Other/Decendant of other	996 (7.5%)		71 (8.0%)	4992 (8.6%)	405 (8.3%)	6464 (8%)
*Income*						
Low	3633 (27.4%)		165 (18.6%)	14,076 (24.3%)	902 (18.4%)	18,776 (24%)
Middle	6740 (50.8%)		380 (42.9%)	28,365 (49.1%)	2206 (45.1%)	37,691 (49%)
High	2895 (21.8%)		341 (38.5%)	15,367 (26.6%)	1783 (36.5%)	20,386 (27%)
*Education*						
Basic	6116 (46.1%)		333 (37.6%)	25,786 (44.6%)	1942 (39.7%)	34,177 (44%)
High school or vocational	4964 (37.4%)		406 (45.8%)	24,142 (41.8%)	2243 (45.9%)	31,755 (41%)
Higher	1394 (10.5%)		133 (15.0%)	6050 (10.5%)	591 (12.1%)	8168 (11%)
Unknown	794 (6.0%)		14 (1.6%)	1830 (3.2%)	115 (2.4%)	2753 (4%)
*Diabetes duration*						
< 1 year	5519 (41.6%)		490 (55.3%)	22,618 (39.1%)	1588 (32.5%)	30,215 (39%)
1–3 years	1452 (10.9%)		36 (4.1%)	7010 (12.1%)	180 (3.7%)	8678 (11%)
3–5 years	1111 (8.4%)		50 (5.6%)	5322 (9.2%)	343 (7.0%)	6826 (9%)
5–7 years	929 (7.0%)		47 (5.3%)	4164 (7.2%)	373 (7.6%)	5513 (7%)
> 7 years	4257 (32.1%)		263 (29.7%)	18,694 (32.3%)	2407 (49.2%)	25,621 (33%)
*Pharmacotherapy*						
Insulin	3082 (23.2%)		239 (27.0%)	12,504 (21.6%)	1950 (39.9%)	17,775 (23%)
Metformin	8820 (66.5%)		505 (57.0%)	45,908 (79.4%)	3859 (78.9%)	59,092 (77%)
Dipeptidyl peptidase-4 inhibitors	1009 (7.6%)		62 (7.0%)	5366 (9.3%)	401 (8.2%)	6838 (9%)
Sulfonylureas	2127 (16.0%)		78 (8.8%)	9339 (16.2%)	748 (15.3%)	12,292 (16%)
Other glucose-lowering drugs	64 (0.5%)		3 (0.3%)	274 (0.5%)	22 (0.4%)	363 (0.5%)
Other lipid-lowering drugs	666 (5.0%)		105 (11.9%)	2542 (4.4%)	368 (7.5%)	3681 (5%)
Beta-blockers	5979 (45.1%)		433 (48.9%)	34,000 (58.8%)	3094 (63.3%)	43,506 (57%)
Calcium channel blockers	3881 (29.3%)		209 (23.6%)	23,081 (39.9%)	1940 (39.7%)	29,111 (38%)
Renin-angiotensin system inhibitors	7683 (57.9%)		612 (69.1%)	42,246 (73.1%)	4017 (82.1%)	54,559 (71%)
Thiazides, thiazide-like drugs	3804 (28.7%)		261 (29.5%)	21,407 (37.0%)	1848 (37.8%)	27,320 (36%)
Loop diuretics	5738 (43.2%)		400 (45.1%)	19,960 (34.5%)	1949 (39.8%)	28,047 (36%)
Potassium-sparing diuretics	2121 (16.0%)		234 (26.4%)	7820 (13.5%)	973 (19.9%)	11,148 (15%)
Nitrates	1419 (10.7%)		81 (9.1%)	10,439 (18.1%)	877 (17.9%)	12,816 (17%)
Vitamin K antagonists	2116 (15.9%)		105 (11.9%)	7914 (13.7%)	578 (11.8%)	10,713 (14%)
Direct oral anticoagulants	1237 (9.3%)		118 (13.3%)	3363 (5.8%)	369 (7.5%)	5087 (7%)
Antiplatelets	7286 (54.9%)		392 (44.2%)	47,515 (82.2%)	4022 (82.2%)	59,215 (77%)
Beta-2 agonist ihalants	2379 (17.9%)		173 (19.5%)	9018 (15.6%)	807 (16.5%)	12,377 (16%)
Anticholinergic inhalants	1345 (10.1%)		77 (8.7%)	4931 (8.5%)	389 (8.0%)	6742 (9%)
Glucocorticoid inhalants	1696 (12.8%)		109 (12.3%)	6357 (11.0%)	559 (11.4%)	8721 (11%)
Antidepressants	1980 (14.9%)		80 (9.0%)	7595 (13.1%)	552 (11.3%)	10,207 (13%)
Non-steroid anti-inflammatory drugs	2493 (18.8%)		226 (25.5%)	11,510 (19.9%)	1141 (23.3%)	15,370 (20%)
Glucocorticoids with systemic action	1783 (13.4%)		92 (10.4%)	5600 (9.7%)	379 (7.7%)	8214 (11%)
*Medical history*						
Nephropathy	3146 (23.7%)		194 (21.9%)	11,126 (19.2%)	1209 (24.7%)	15,675 (20%)
Neuropathy	1563 (11.8%)		88 (9.9%)	6260 (10.8%)	608 (12.4%)	8519 (11%)
Retinopathy	1630 (12.3%)		94 (10.6%)	7484 (12.9%)	901 (18.4%)	10,109 (13%)
Hypertension	7654 (57.7%)		481 (54.3%)	36,476 (63.1%)	3219 (65.8%)	47,830 (62%)
Chronic obstructive pulmonary disease	2367 (17.8%)		128 (14.4%)	8046 (13.9%)	644 (13.2%)	11,185 (15%)
Atrial fibrillation	4119 (31.0%)		213 (24.0%)	12,303 (21.3%)	948 (19.4%)	17,583 (23%)
Venous thromboembolism	991 (7.5%)		75 (8.5%)	3046 (5.3%)	261 (5.3%)	4373 (6%)
*Laboratory data*						
Hemoglobin A1c, mmol/mol	50 [44; 59]		50 [39; 65]	50 [45; 57]	54 [46; 66]	50 [45; 58]
LDL cholesterol, mmol/L	2.7 [2.1; 3.3]		2.7 [2.1; 3.3]	1.8 [1.4; 2.3]	1.7 [1.4; 2.2]	1.9 [1.5; 2.5]
Estimated GFR, mL/min per 1.73 m2	72 [52; 89]		83 [64; 97]	75 [56; 90]	82 [63; 95]	75 [56; 90]
Eligibility						
*Cardiovascular disease*	13,268 (100%)		886 (100%)	57,808 (100%)	4891 (100%)	76,853 (100%)
Myocardial infarction	3031 (22.8%)		234 (26.4%)	21,613 (37.4%)	1880 (38.4%)	29,758 (39%)
Stroke	4539 (34.2%)		205 (23.1%)	18,002 (31.1%)	1115 (22.8%)	23,861 (31%)
Coronary revascularization	2359 (17.8%)		204 (23.0%)	26,271 (45.5%)	2433 (49.7%)	31,267 (41%)
Peripheral arterial disease	2496 (18.8%)		134 (15.1%)	11,642 (20.1%)	900 (18.4%)	15,172 (20%)
Heart failure	5454 (41.1%)		411 (46.4%)	16,203 (28.0%)	1666 (34.1%)	23,734 (31%)

Baseline characteristics for the time-varying cohort. Continuous variables are reported as mean (standard deviation) or median [interquartile range], and categorical variables as count (percentage). LDL cholesterol in mg/dl is mmol/L multiplied by 38.67. Hemoglobin A1c in % is (mmol/mol multiplied by 0.095) + 2.15. *LDL*: low-density lipoprotein; *GFR*: glomerular filtration rate

### Sodium glucose cotransporter-2 inhibitor or glucagon-like peptide-1 receptor agonist in combination with statin for secondary prevention

**Fig. 1:**
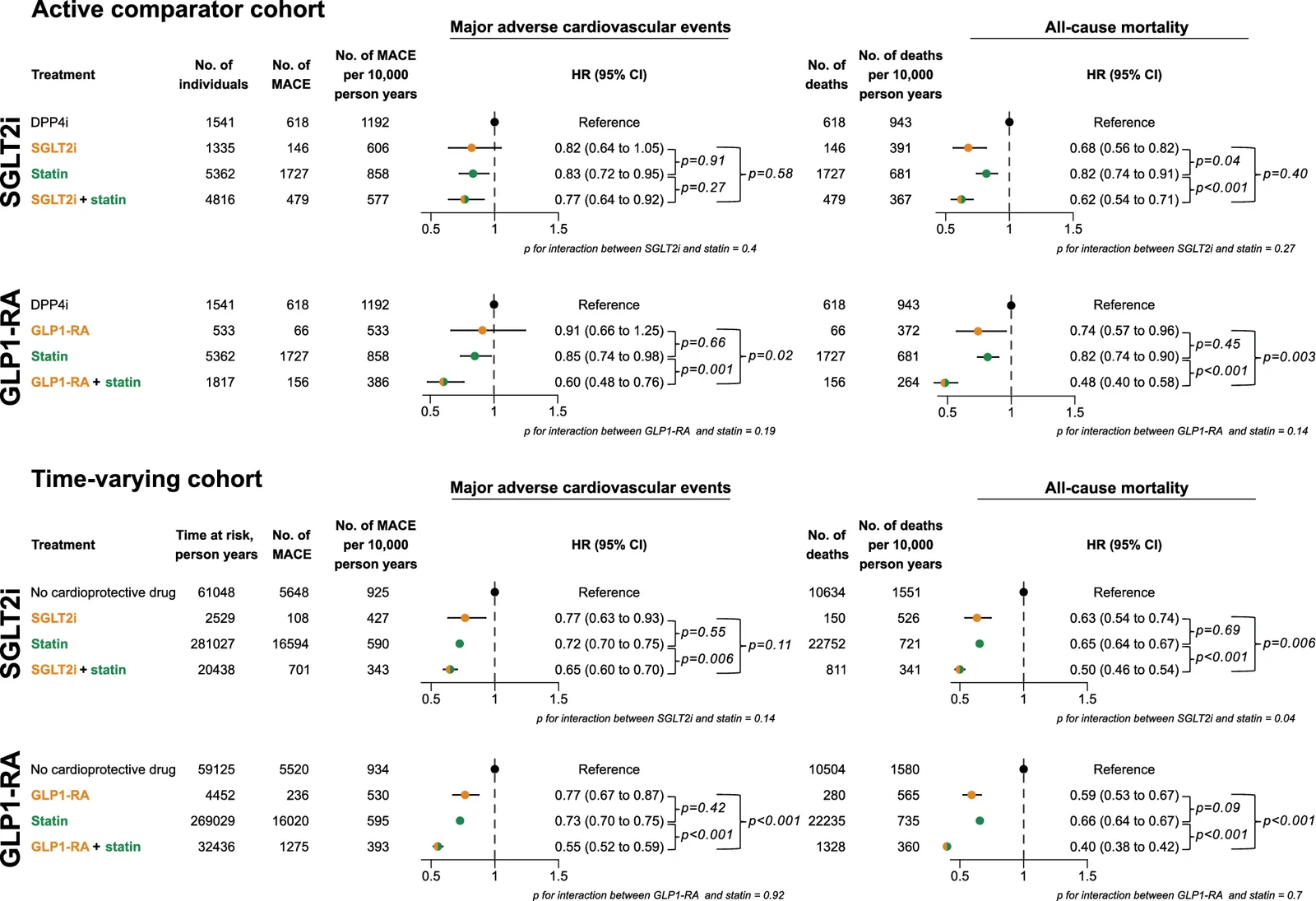
Association of sodium glucose cotransporter-2 inhibitor, glucagon-like peptide-1 receptor agonist, and statin with major adverse cardiovascular events and all-cause mortality. Multivariable-adjusted Cox proportional hazards model estimates for secondary MACE and all-cause mortality for sodium-glucose cotransporter-2 inhibitor (upper section of upper and lower panels) and glucagon-like peptide-1 receptor agonist (lower sections of upper and lower panels) for the active comparator and time-varying cohorts. The models are adjusted for covariates listed in the baseline characteristics tables, plus sodium-glucose cotransporter-2 inhibitor in the glucagon-like peptide-1 receptor agonist models, and vice versa. No cardioprotective drug denotes no use of statin or cardioprotective glucose-lowering drug. Abbreviations: *MACE*: Major adverse cardiovascular events; *HR*: Hazard ratio; *CI* Confidence interval; *DPP4i*: Dipeptidyl peptidase-4 inhibitor; *SGLT2i*: Sodium-glucose cotransporter 2 inhibitor; *GLP-1RA*: Glucagon-like peptide-1 receptor agonist

When evaluated individually in multivariable adjusted models, both sodium-glucose cotransporter-2 inhibitors and glucagon-like peptide-1 receptor agonists were associated with the lowest risk of secondary MACE and all-cause mortality when used in combination with statins (Fig. 1). The stepwise reduction in risk observed with combined therapy, relative to either drug alone, was more pronounced for glucagon-like peptide-1 receptor agonists (Fig. 1, lower section of upper and lower panels). Interaction tests between the specific cardioprotective glucose-lowering drug and statin yielded *p*-values > 0.05 across all models, with the exception of all-cause mortality in the time-varying cohort for sodium-glucose cotransporter-2 inhibitors (*p* = 0.04; Fig. 1, upper section of lower panel).

### Combination of cardioprotective glucose-lowering drug and statin for secondary prevention

**Fig. 2:**
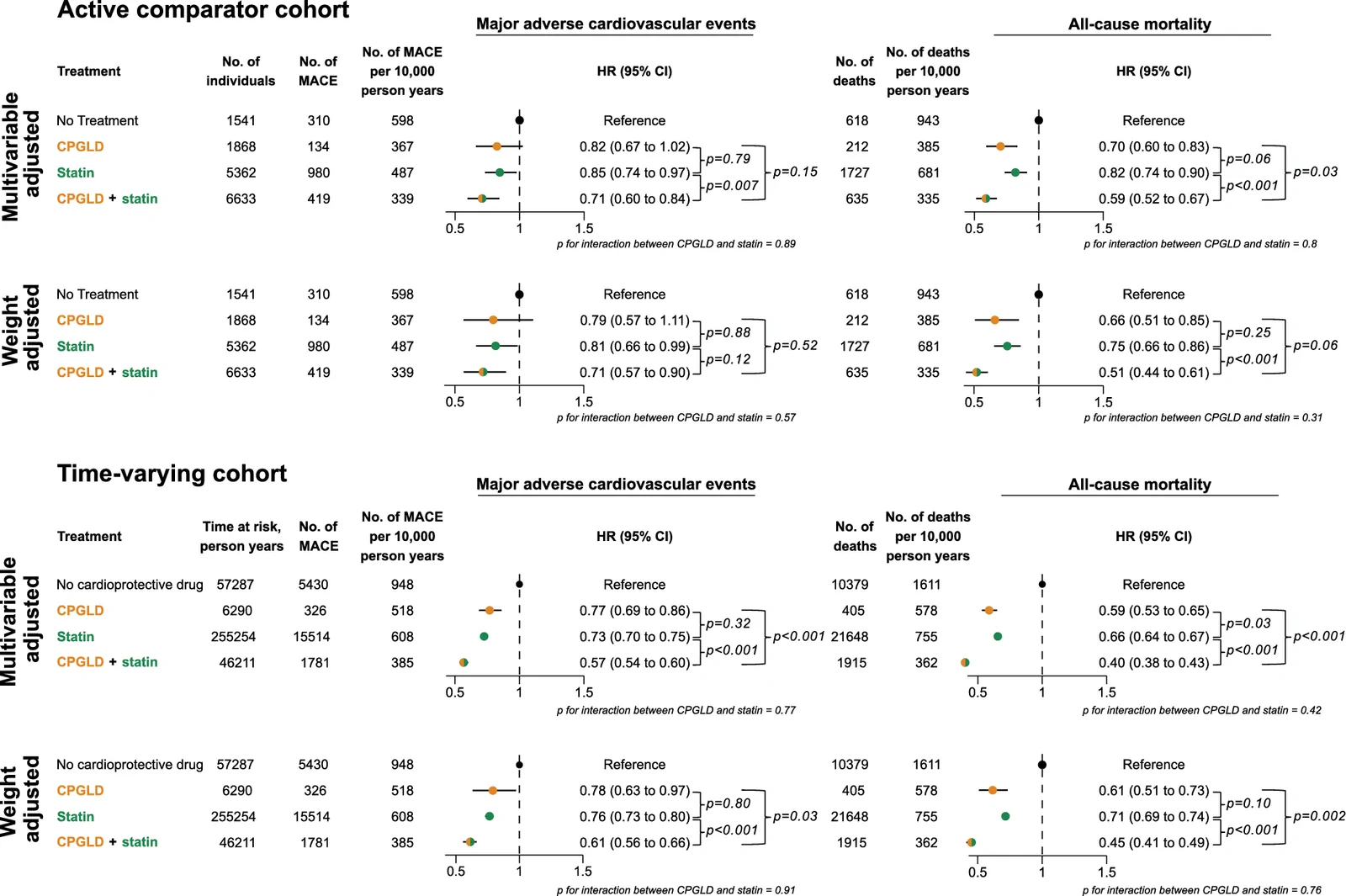
Association of cardioprotective glucose-lowering drug and statin with major adverse cardiovascular events and all-cause mortality. Multivariable-adjusted Cox proportional hazards model estimates for secondary MACE and all-cause mortality adjusted for covariates in the baseline characteristics tables for the active comparator and the time-varying cohorts (upper panels), and weight-adjusted Cox model estimates after balancing baseline covariates as seen in Supplementary Figure S3 for the active comparator cohort and Supplementary Figures S4-S12 for the time-varying cohort using inverse-probability of treatment weighting (lower panels). Cardioprotective glucose-lowering drug is either sodium-glucose cotransporter-2 inhibitor or glucagon-like peptide-1 receptor agonist. No cardioprotective drug denotes no use of statin or cardioprotective glucose-lowering drug. Abbreviations: *MACE*: Major adverse cardiovascular events; *HR*: Hazard ratio; *CI* Confidence interval; *DPP4i*: Dipeptidyl peptidase-4 inhibitor; *CPGLD*: Cardioprotective glucose-lowering drug

When pooling sodium-glucose cotransporter-2 inhibitors and glucagon-like peptide-1 receptor agonists into a single category of cardioprotective glucose-lowering drugs in multivariable adjusted models to obtain maximal statistical power for further analyses, combined treatment with a statin was also associated with the lowest risk of secondary MACE and all-cause mortality. In the active comparator cohort with secondary MACE as outcome, hazard ratios with dipeptidyl peptidase-4 inhibitor as reference were 0.82 (95% confidence interval (CI): 0.67 to 1.02) for cardioprotective glucose lowering drug alone, 0.85 (0.74 to 0.97) for dipeptidyl peptidase-4 inhibitor and statin combined, and 0.71 (0.60 to 0.84) for cardioprotective glucose-lowering drug and statin combined (Fig. 2, Active comparator cohort, upper panel). Corresponding hazard ratios in the time-varying cohort with no cardioprotective glucose lowering drug or statin as reference were 0.77 (0.69 to 0.86) for cardioprotective glucose lowering drug alone, 0.73 (0.70 to 0.75) for statin alone, and 0.57 (0.54 to 0.60) for cardioprotective glucose lowering drug and statin combined (Fig. 2, Time-varying cohort, upper panel).

In the active comparator cohort with all-cause mortality as outcome, hazard ratios with dipeptidyl peptidase-4 inhibitor as reference were 0.70 (95% CI 0.60 to 0.83) for cardioprotective glucose lowering drug alone, 0.82 (0.74 to 0.90) for dipeptidyl peptidase-4 inhibitor and statin combined, and 0.59 (0.52 to 0.67) for cardioprotective glucose lowering drug and statin combined (Fig. 2, Active-comparator cohort, upper panel). Corresponding hazard ratios in the time-varying cohort with no cardioprotective glucose lowering drug or statin as reference were 0.59 (0.53 to 0.65) for cardioprotective glucose lowering drug alone, 0.66 (0.64 to 0.67) for statin alone, and 0.40 (0.38 to 0.43) for cardioprotective glucose lowering drug and statin combined (Fig. 2, Time-varying cohort, upper panel).

Inverse probability of treatment weighted models yielded similar patterns as in main analyses (Fig. 2, Active comparator cohort and Time-varying cohort, lower panels). Weighting effectively balanced covariates in both cohorts (Supplementary Figures S3–S12) with slight deviation at some time points in the time-varying cohort; however, additional adjustments for these variables yielded similar results (Supplementary Figure S13).

In both cohorts *p* for interaction between cardioprotective glucose-lowering drug and statin was > 0.05 for MACE and all-cause mortality (Fig. 2).

Thus, compared to the reference group (no use of statins or cardioprotective glucose-lowering drug) in both cohorts, the use of a cardioprotective glucose-lowering drug alone, a statin alone, or combined treatment were all associated with a lower risk of secondary MACE and all-cause mortality. Except for cardioprotective glucose-lowering drug alone in the active comparator cohort, all upper 95% CIs were below 1.00 in all analyses.

**Fig. 3:**
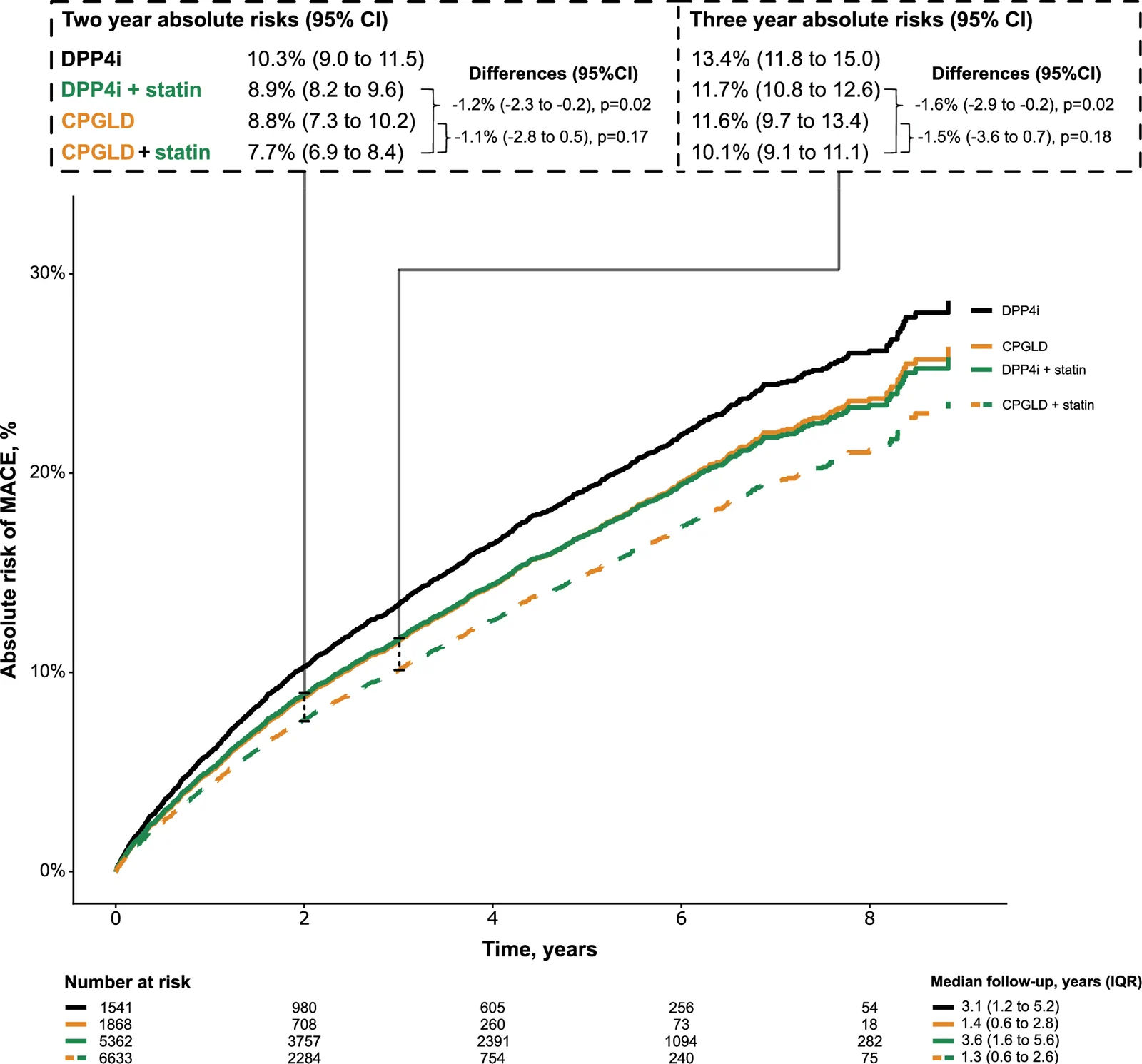
Cumulative incidence of major adverse cardiovascular events with use of cardioprotective glucose-lowering drug and statin. Cause-specific cumulative incidence of secondary MACE for the active comparator cohort adjusted for covariates listed in the baseline characteristics table by standardization through the G-formula. Death due to noncardiovascular causes was treated as competing risk. Cardioprotective glucose-lowering drug is either sodium-glucose cotransporter-2 inhibitor or glucagon-like peptide-1 receptor agonist. Abbreviations: *MACE*: Major adverse cardiovascular events; *DPP4i*: Dipeptidyl peptidase-4 inhibitor; *CPGLD*: Cardioprotective glucose-lowering drug; *CI* Confidence interval; *IQR*: Interquartile range

Two-year absolute risk differences were − 1.1% (95% CI − 2.8% to 0.5%, *p* = 0.17) for combined treatment versus cardioprotective glucose-lowering drug alone and − 1.2% ( − 2.3% to − 0.2%, *p* = 0.02) for combined treatment versus statin alone (Fig. 3). Corresponding three-year absolute risk differences of secondary MACE were − 1.5% (95% CI − 3.6% to 0.7%, *p* = 0.18) and − 1.6% ( − 2.9% to − 0.2%, *p* = 0.02), respectively. Median follow-up for the MACE outcome in each group was 3.1 years (interquartile range: 1.2 to 5.2 years) for dipeptidyl peptidase-4 inhibitor, 3.6 years (1.6 to 5.6 years) for dipeptidyl peptidase-4 inhibitor and statin, 1.4 years (0.6 to 2.8 years) for cardioprotective glucose-lowering drug, and 1.3 years (0.6 to 2.6 years) for cardioprotective glucose-lowering drug and statin (Fig. 3). Supplementary Figure S14 shows the cumulative incidence curves with confidence intervals plotted.

### Subgroup analyses

**Fig. 4:**
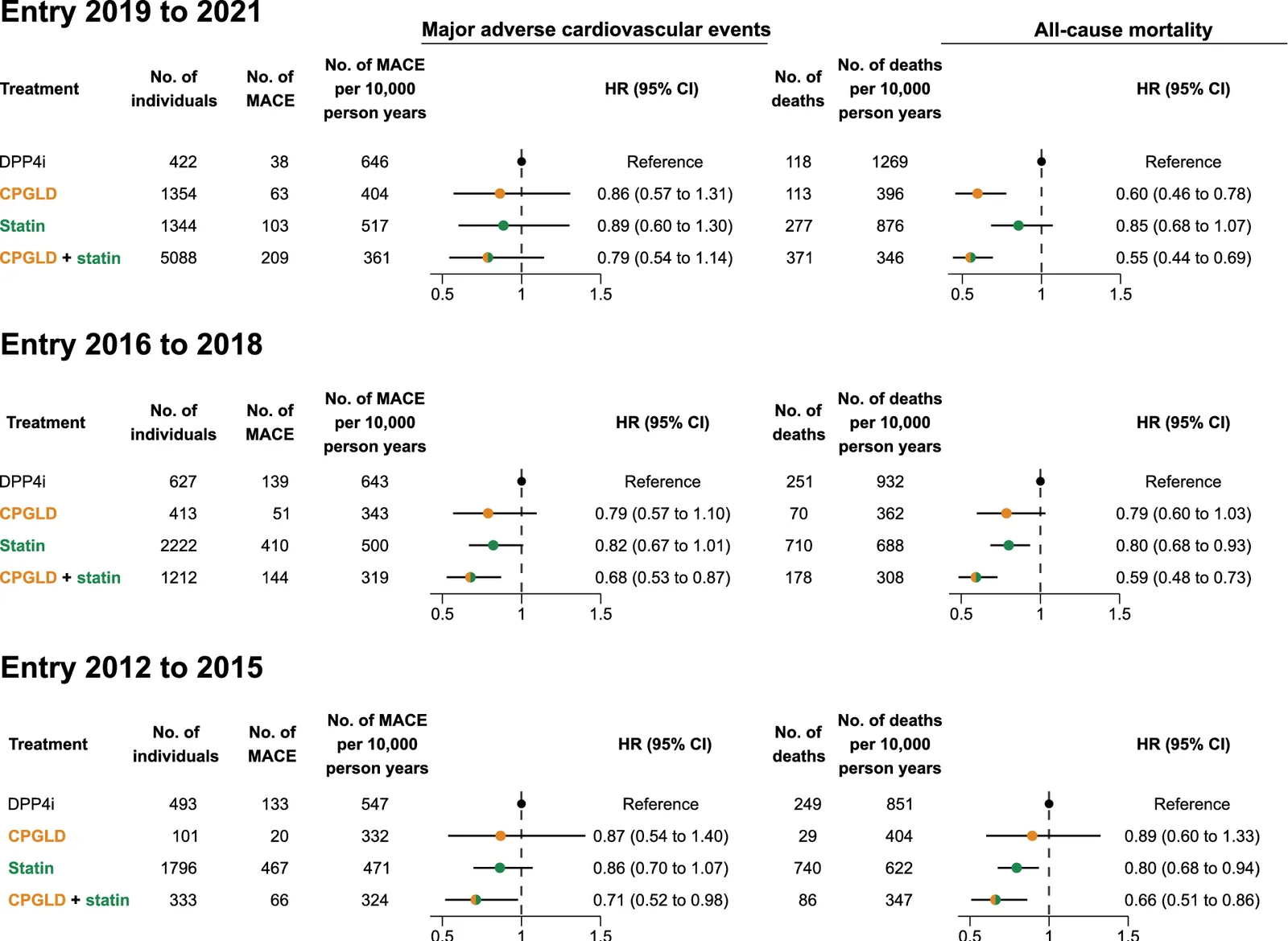
Association of cardioprotective glucose-lowering drug and statin with major adverse cardiovascular events and all-cause mortality by year of entry. Multivariable-adjusted Cox proportional hazards model estimates for secondary MACE and all-cause mortality adjusted for covariates in the baseline characteristics table for the active comparator cohort, when stratified by year of entry into observation (2019–2021, 2016–2018, and 2012–2015). Cardioprotective glucose-lowering drug is either sodium-glucose cotransporter-2 inhibitor or glucagon-like peptide-1 receptor agonist. No cardioprotective drug denotes no use of statin or cardioprotective glucose-lowering drug. Abbreviations: *MACE*: Major adverse cardiovascular events; *HR*: Hazard ratio; *CI* Confidence interval; *DPP4i*: Dipeptidyl peptidase-4 inhibitor; *CPGLD*: Cardioprotective glucose-lowering drug

**Fig. 5:**
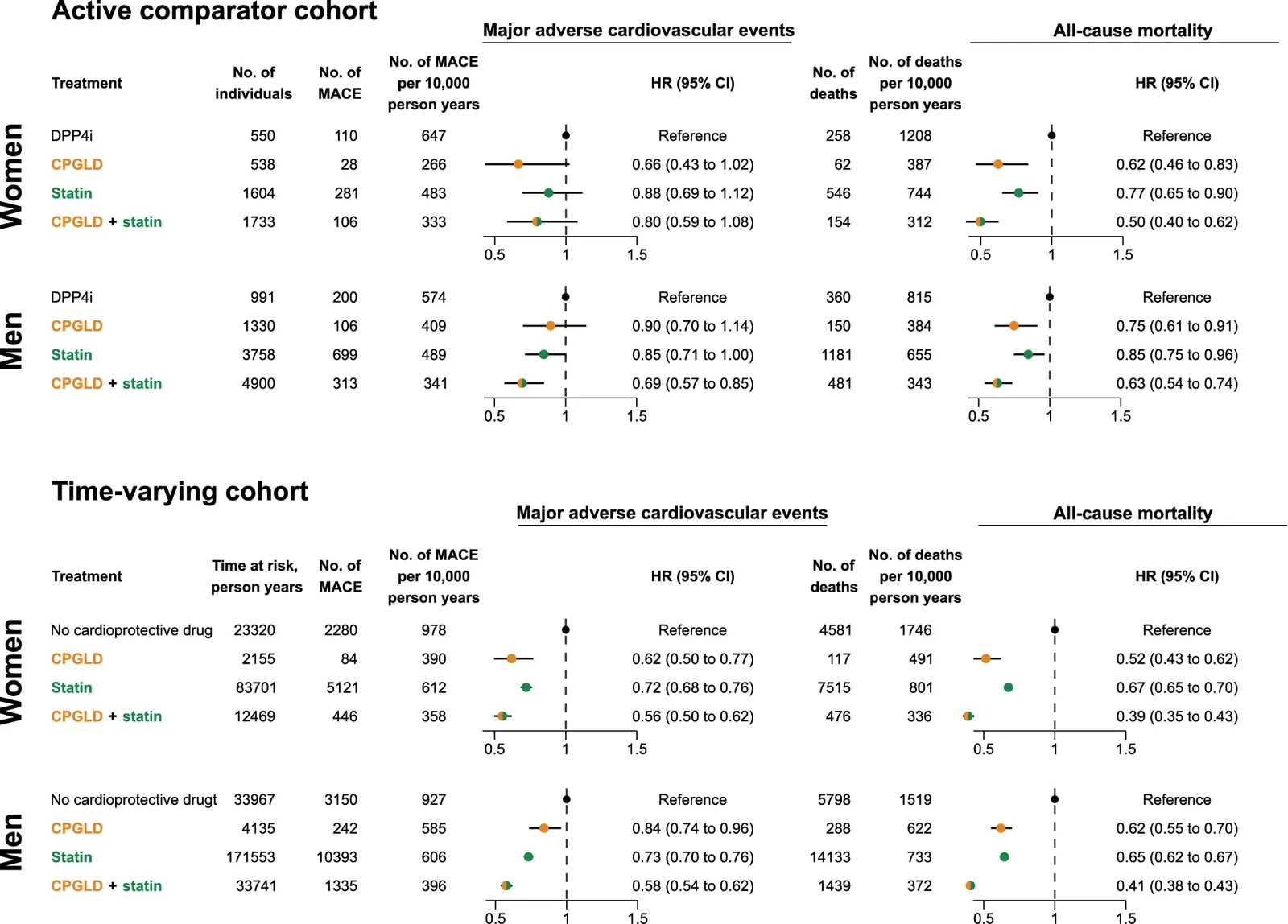
Association of cardioprotective glucose-lowering drug and statin with major adverse cardiovascular events and all-cause mortality by sex. Multivariable-adjusted Cox proportional hazards model estimates for secondary MACE and all-cause mortality adjusted for covariates in the baseline characteristics tables for the active comparator and the time-varying cohorts, when stratified by women (upper section of upper and lower panels) and men (lower section of upper and lower panels). Cardioprotective glucose-lowering drug is either sodium-glucose cotransporter-2 inhibitor or glucagon-like peptide-1 receptor agonist. No cardioprotective drug denotes no use of statin or cardioprotective glucose-lowering drug. Abbreviations: *MACE*: Major adverse cardiovascular events; *HR*: Hazard ratio; *CI* Confidence interval; *DPP4i*: Dipeptidyl peptidase-4 inhibitor; *CPGLD*: Cardioprotective glucose-lowering drug

**Fig. 6:**
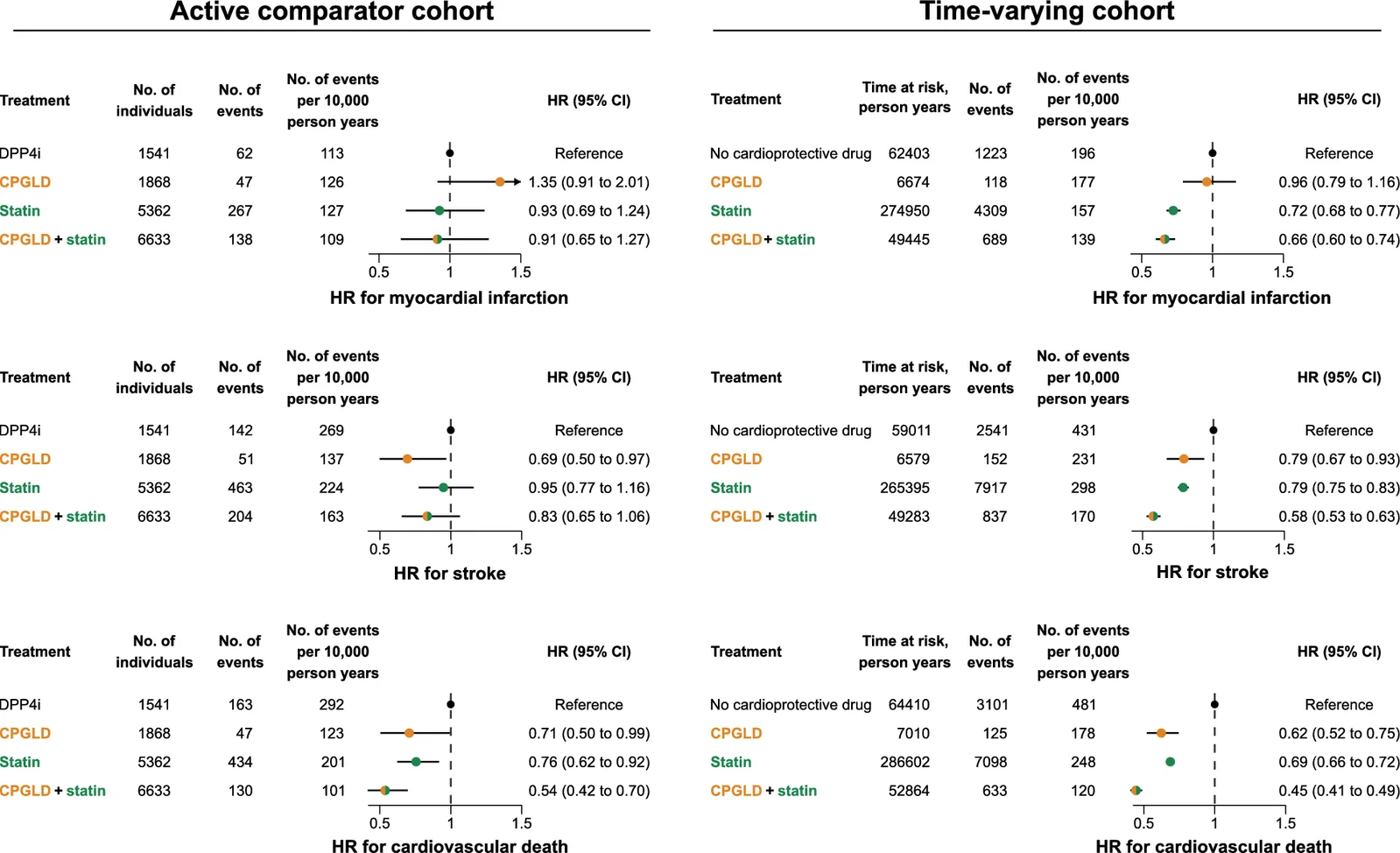
Association of cardioprotective glucose-lowering drug and statin with nonfatal myocardial infarction, stroke, and cardiovascular death. Multivariable-adjusted Cox proportional hazards model estimates for nonfatal myocardial infarction, stroke, and cardiovascular death adjusted for covariates in the baseline characteristics tables for the active comparator (left column) and the time-varying (right column) cohorts. Cardioprotective glucose-lowering drug is either sodium-glucose cotransporter-2 inhibitor or glucagon-like peptide-1 receptor agonist. No cardioprotective drug denotes no use of statin or cardioprotective glucose-lowering drug. Abbreviations: *HR*: Hazard ratio; *CI* Confidence interval; *DPP4i*: Dipeptidyl peptidase-4 inhibitor; *CPGLD*: Cardioprotective glucose-lowering drug

In analyses stratified by year of entry (2019–2021, 2016–2018, and 2012–2015), combined treatment remained associated with the lowest hazard ratios of secondary MACE and all-cause mortality across all strata (Fig. 4 and Supplementary Figure S15). In analyses stratified by sex, combined treatment was associated with the lowest hazard ratios in all analyses, except for secondary MACE among women in the active comparator cohort (Fig. 5). However, confidence intervals were large for some analyses in women and *p* for interaction was > 0.05. Results for individual subcomponents of MACE, that is, myocardial infarction, stroke, and cardiovascular death, showed considerable variability in the active comparator cohort. However, overall patterns were largely consistent with the main results when considering all six analyses together (Fig. 6).

### Sensitivity analyses

In all sensitivity analyses, findings remained consistent with the primary models. Estimates for secondary MACE were stable across Cox models adjusted only for age, sex, and ethnicity, adjusted further for general demographic variables, and for the fully adjusted models (Supplementary Table S7). Adding hemoglobin A1c, estimated glomerular filtration rate, and low-density lipoprotein cholesterol to the adjustments also resulted in similar stepwise risk reduction with either drug alone to combined treatment (Supplementary Figure S16).

Estimates for secondary MACE and all-cause mortality were stable within strata of statin treatment duration prior to index (< 1 year, 1–3 years, 3–5 years, > 5 years) (Supplementary Figures S17–S18). When statin use in the time-varying cohort was restricted to a proportion of days covered > 80% in the preceding year (thereby reclassifying non-adherent users as non-users), combined treatment remained associated with the lowest risk of secondary MACE and all-cause mortality (Supplementary Figure S19). Analyses restricted to individuals on optimal baseline treatment (antiplatelet and renin-angiotensin system inhibitors) yielded results similar to the main model (Supplementary Figure S20).

Extending the grace period for classifying a diagnosis of new myocardial infarction or stroke from 30 to 90 days slightly lowered the absolute number of events, but multivariable adjusted Cox estimates were similar to the main model (Supplementary Figure S21). Restricting the cohort to individuals with a prior myocardial infarction demonstrated that the associated risk reductions in MACE were largely driven by a lower risk of cardiovascular death, consistent with the full cohort (Supplementary Figure S22). Furthermore, Fine and Gray competing risk models for secondary MACE accounting for noncardiovascular death were comparable to the multivariable adjusted Cox results across all drug classes and stratifications (Supplementary Figures S23-S25).

## Discussion

In a nationwide cohort of individuals with type 2 diabetes and established cardiovascular disease, combined use of a cardioprotective glucose-lowering drug and a statin was associated with lower risk of MACE than using either drug alone. This is consistent with and reinforces existing clinical trial evidence demonstrating independent benefits of each therapy that has led international guidelines to recommend the combined use of cardioprotective glucose-lowering drugs and statins for secondary prevention of MACE in individuals with type 2 diabetes.

To our knowledge, we are the first to explore cardioprotective glucose-lowering drugs and statins individually and combined in individuals with diabetes and established cardiovascular disease. In support of our findings, a recent systematic review and meta-analysis using data from randomized intervention trials indicated that the benefits to several cardiovascular outcomes was independent of baseline lipid-lowering drug use [38]. The analysis was limited by only few studies reporting these stratified outcomes.

Although an absolute risk of 10–13.5% over three years is high, it is consistent with rates observed in contemporary trials for glucose-lowering drugs in secondary prevention [4, 16]. Because this study focuses on individuals with established cardiovascular disease, applying these findings to lower-risk populations may be inappropriate. Instead, a distinction may be warranted for primary versus secondary prevention, as a recent study by our group found in individuals with diabetes but no history of cardiovascular diseases, that statins were associated with lowest risk of first time atherosclerotic cardiovascular disease [39]. Addition of a cardioprotective glucose-lowering drug was associated with little added benefit.

Guideline recommendations for statins, antiplatelets, renin-angiotensin system inhibitors, and cardioprotective glucose-lowering drugs in secondary prevention of MACE were established before or during the observation period, so their uptake seems low in both cohorts in the present study. Nevertheless, studies from Europe and the United States have demonstrated this delayed initiation of cardioprotective therapies in individuals with type 2 diabetes, including those with established cardiovascular disease in real-world practice [18, 19]. Similarly, attainment of lipid-lowering treatment goals among high-risk individuals remains suboptimal [20, 40–42], and initiation of newer glucose-lowering therapies may vary according to patients’ socioeconomic characteristics [43]. Our results showed lowest risk of secondary MACE associated with combined use of cardioprotective glucose-lowering drugs and statins, and a sensitivity analysis comparing individuals who were already on optimal therapy (antiplatelet and renin-angiotensin system inhibitor treatment) showed similar results to the main analysis. In this context, our findings support guidelines that recommend multiple cardioprotective drugs for individuals with type 2 diabetes to achieve maximal protection from secondary cardiovascular events.

Strengths of our study include a large nationwide cohort with up to ten years of follow-up, negligible losses to follow-up, and near complete data for included covariates, enabling us to also investigate the interactions of cardioprotective glucose-lowering drugs and statins. Further, results were robust across two designs accounting for different sources of bias. Lastly, our results add to evidence reported in clinical trials by exploring a question that is unlikely to be studied in future trials due to ethical concerns of not using these drugs in high-risk individuals with diabetes.

Study limitations include the use of prescriptions to define type 2 diabetes, which may exclude individuals managed with lifestyle intervention alone, not yet initiated on glucose-lowering therapy. However, it is unlikely that many with both hyperglycemia and established cardiovascular disease remain untreated with pharmacotherapy. Furthermore, prescriptions have yielded 95% positive predictive value for diabetes diagnoses previously in Danish registers [44]. Second, a limitation of the design is its vulnerability to immortal time bias in statin groups (in the form of prevalent use of statin on index date with potential depletion of susceptibles). This is impossible to avoid in real-world data, as patients frequently initiate combination therapy sequentially rather than simultaneously, making it unfeasible to assign index dates based on initiation of both drug classes on the same calendar day. Existing use of statin may introduce bias if individuals at highest risk experience early events and are subsequently excluded from the statin groups in the cohort. However, sensitivity analyses stratified by the duration of statin use suggested that this was not a major concern. In continuation, the similarity between models which included only fixed characteristics (age, sex, and descent), and the fully adjusted models, suggested that, if present, the magnitude of potential collider bias that prevalent use of statin could introduce, would not be expected to alter the conclusions. Third, covariates that were accessible through registers were not as detailed as would be collected in a clinical trial and important covariates such as smoking, body mass index, and urinary albumin/creatinine could not be assessed directly. Alternatively, we included demographic, medical history, and pharmacotherapy variables, to serve as proxies for intermediate health related and biochemical covariates, although they may not fully capture the clinical reality influencing treatment decisions. The similarity of effect estimates between parsimonious models and the fully adjusted model may suggest that key sources of imbalance across treatment groups lay in measured general demographic factors such as sex, age, descent, socioeconomic status, and diabetes duration, but it cannot be ruled out that other important unmeasured confounders that influence access to healthcare, adherence to preventive care, and more aggressive cardiovascular risk management overall were inadequately controlled for. Fourth, during the observation period treatment guidelines, especially for cardioprotective glucose-lowering drugs, have been updated several times. Indeed, the uptake of sodium-glucose cotransporter-2 inhibitors and glucagon-like peptide-1 receptor agonists increased dramatically during the later years of the study period, as exemplified by shorter median follow-up in the groups initiating cardioprotective glucose-lowering drugs [22]. This creates the possibility that uneven follow-up times, improvements in cardiovascular standards of care, evolving guidelines, and changes in diagnostic practices over time may contribute as confounders driving to the observed differences in outcomes. Nevertheless, our sensitivity analyses suggest that they were not key drivers of the results: stratifying analyses by key periods of entry into observation (2019–2021, 2016–2018, and 2012–2015) did not result in any major modification of the stepwise associated risk reductions. Also, in the time-varying cohort, which inherently updated covariates and treatment status yearly in parallel with altered standards of care, showed similar results as the active comparator cohort, and weighting achieved good balance in both cohorts for all covariates, including the year of entry, and results remained similar. Despite these measures, and adjustments for year of entry in all the main models, some residual confounding by secular trends cannot be ruled out entirely. Fifth, despite using several designs and approaches to address potential biases, residual confounding by indication and healthcare utilization is always a possibility in such observational studies. Also, as information on actual use of medication is not available, studies such as ours must rely on relatively coarse classifications based on prescription registers, which may result in misclassifications. As a result, conclusions based solely on register-based observational data should remain appropriately cautious, particularly with respect to causal interpretation. Finally, while the project outline, the cohort, and hypothesis was predefined as part of a PhD project [45], a full protocol has not been published for this specific study, as there is a lack of consensus for publishing such protocols for observational studies. Thus, this could be considered a limitation shared with most observational studies.

## Conclusion

In individuals with type 2 diabetes and established cardiovascular disease, treatment with a cardioprotective glucose-lowering drug and statin in combination was associated with lower risk of MACE than using either drug alone. Some biases can never be completely excluded in real-world pharmacotherapy use studies such as this one, including confounding by indication, time-related or immortal time biases, and shifting standards of care, rendering causal interpretation unattainable; however, our results seemed consistent across numerous sensitivity analyses and designs.

## Supplementary Information

Additional file 1 (DOCX 8725 KB)

## Data Availability

The datasets generated and/or analyzed during the current study are not publicly available due privacy laws in Denmark. The data are held on secure servers managed by Statistics Denmark ([https://www.dst.dk/]). Access to the databases may be granted to researchers affiliated to publicly funded Danish research and analyses institutions conditional on having an application accepted by Statistics Denmark.

## Abbreviations

ICD: International Classification of Diseases
ISCED: International Standard Classification of Education
MACE: Major adverse cardiovascular event
OECD: Organization for Economic Cooperation and Development
