# Supplementary material for: Cardioprotective glucose-lowering drugs, statins, and secondary major adverse cardiovascular events: a nationwide cohort study of individuals with type 2 diabetes and cardiovascular disease
Source: Cardiovasc Diabetol. 2026 Jun 16;25:254. doi: 10.1186/s12933-026-03242-9 (PMC13501814; doi:10.1186/s12933-026-03242-9)
Supplement: Supplementary file 1 [file 12933_2026_3242_MOESM1_ESM.docx]

**Supplemental Appendix:**

Cardioprotective glucose-lowering drugs, statins, and secondary major adverse cardiovascular events: A nationwide cohort study of individuals with type 2 diabetes and cardiovascular disease

Tummas Ternhamar, MD^1,2,3^, Mia Ø. Johansen, PhD^2,3,4^, Børge G. Nordestgaard, Professor ^1,2,3^, Shoaib Afzal, MD, Professor^1,2,3^

^1^Department of Clinical Biochemistry, Copenhagen University Hospital - Herlev and Gentofte, Borgmester Ib Juuls Vej 73, DK-2730, Herlev, Denmark, ^2^The Copenhagen General Population Study, Copenhagen University Hospital - Herlev and Gentofte, Borgmester Ib Juuls Vej 73, DK-2730, Herlev, Denmark, ^3^Department of Clinical Medicine, Faculty of Health and Medical Sciences, University of Copenhagen, Blegdamsvej 3, DK-2200, Copenhagen, Denmark, ^4^Department of Clinical Biochemistry, Copenhagen University Hospital - Rigshospitalet, Blegdamsvej 9, DK-2100, Copenhagen, Denmark.

Correspondence:

Shoaib Afzal, MD, PhD, DMSc, Professor, Chief Physician

Department of Clinical Biochemistry

Copenhagen University Hospital - Herlev and Gentofte

Borgmester Ib Juuls Vej 73

Elevator 7, 4th Floor, N5

DK-2730 Herlev, Denmark

Phone: +45 38 68 38 40

E-mail: Shoaib.Afzal@regionh.dk

**Table of contents**

[Supplementary Methods	4](#_Toc228664119)

[Supplementary Table S1: Exposure/treatment definitions	5](#_Toc228664120)

[Supplementary Table S2: Major adverse cardiovascular event outcome definitions	6](#_Toc228664121)

[Supplementary Table S3: Other pharmacotherapy and medical diagnosis definitions	7](#_Toc228664122)

[Supplementary Table S4: Laboratory variables	9](#_Toc228664123)

[Supplementary Table S5: Covariate rationale and collider assessment	10](#_Toc228664124)

[Supplementary Table S6: Sizes of treatment groups at each timepoint in the time-varying cohort	12](#_Toc228664125)

[Supplementary Table S7: Stepwise adjusted models for the primary outcome	13](#_Toc228664126)

[Supplementary Figure S1: Flowchart for the active comparator cohort	14](#_Toc228664127)

[Supplementary Figure S2: Flowchart for the time-varying cohort	15](#_Toc228664128)

[Supplementary Figure S3: Covariate balances at baseline before and after weighting in the active comparator cohort	15](#_Toc228664129)

[Supplementary Figure S4: Covariate balances at before and after weighting at the first timepoint (V0) in the time-varying cohort	17](#_Toc228664130)

[Supplementary Figure S5: Covariate balances at before and after weighting at the second timepoint (V1) after one year in the time-varying cohort	18](#_Toc228664131)

[Supplementary Figure S6: Covariate balances at before and after weighting at the third timepoint (V2) after two years in the time-varying cohort	19](#_Toc228664132)

[Supplementary Figure S7: Covariate balances at before and after weighting at the fourth timepoint (V3) after three years in the time-varying cohort	21](#_Toc228664133)

[Supplementary Figure S8: Covariate balances at before and after weighting at the fifth timepoint (V4) after four years in the time-varying cohort	23](#_Toc228664134)

[Supplementary Figure S9: Covariate balances at before and after weighting at the sixth timepoint (V5) after five years in the time-varying cohort	25](#_Toc228664135)

[Supplementary Figure S10: Covariate balances at before and after weighting at the seventh timepoint (V6) after six years in the time-varying cohort	27](#_Toc228664136)

[Supplementary Figure S11: Covariate balances at before and after weighting at the eighth timepoint (V7) after seven years in the time-varying cohort	30](#_Toc228664137)

[Supplementary Figure S12: Covariate balances at before and after weighting at the ninth timepoint (V8) after eight years in the time-varying cohort	33](#_Toc228664138)

[Supplementary Figure S13: Weight adjusted model in the time-varying cohort with unbalanced covariates added to the outcome model	36](#_Toc228664139)

[Supplementary Figure S14: Cumulative incidence of major adverse cardiovascular events with use of cardioprotective glucose-lowering drug and statin with confidence intervals	37](#_Toc228664140)

[Supplementary Figure S15: Association of cardioprotective glucose-lowering drug and statin with major adverse cardiovascular events and all-cause mortality by year of entry in the time-varying cohort	38](#_Toc228664141)

[Supplementary Figure S16: Association of cardioprotective glucose-lowering drug and statin with major adverse cardiovascular events and all-cause mortality additionally adjusted for laboratory values	39](#_Toc228664142)

[Supplementary Figure S17: Association of cardioprotective glucose-lowering drug and statin with major adverse cardiovascular events and all-cause mortality by duration of statin treatment in the active comparator cohort	40](#_Toc228664143)

[Supplementary Figure S18: Association of cardioprotective glucose-lowering drug and statin with major adverse cardiovascular events and all-cause mortality by duration of statin treatment in the time-varying cohort	41](#_Toc228664144)

[Supplementary Figure S19: Statin therapy defined as >80% proportion days covered in the preceding year for the time-varying cohort	42](#_Toc228664145)

[Supplementary Figure S20: Association of cardioprotective glucose-lowering drug and statin with major adverse cardiovascular events and all-cause mortality in individuals with antiplatelets and renin-angiotensin system inhibitors at baseline	43](#_Toc228664146)

[Supplementary Figure S21: Association of cardioprotective glucose-lowering drug and statin with major adverse cardiovascular events and all-cause mortality using a 90-day grace period	44](#_Toc228664147)

[Supplementary Figure S22: Association of cardioprotective glucose-lowering drug and statin with nonfatal myocardial infarction, stroke, and cardiovascular death in individuals with prior myocardial infarction	45](#_Toc228664148)

[Supplementary Figure S23: Association of cardioprotective glucose-lowering drugs and statin with major adverse cardiovascular events using Fine and Gray models	46](#_Toc228664149)

[Supplementary Figure S24: Association of cardioprotective glucose-lowering drugs and statin with major adverse cardiovascular events by year of entry using Fine and Gray models	47](#_Toc228664150)

[Supplementary Figure S25: Association of cardioprotective glucose-lowering drugs and statin with major adverse cardiovascular events by sex using Fine and Gray models	48](#_Toc228664151)

[References	49](#_Toc228664152)

# Supplementary Methods

*R-packages*

To compute all Cox proportional hazards models we used the “coxph” function from the “survival” R-package^1^. It can also be extended to include time-varying covariates in a time-dependent Cox analysis^2^. The “adjustedcif” function from the “adjustedCurves” R-package was used to compute standardized cumulative incidence curves using the G-formula^3^. Variance of the standardized cumulative incidence function was estimated using the influence function, assuming asymptotic normality variance. To compute pairwise differences in 3-year absolute risks we applied the “adjusted_curve_diff” function also implemented in the “adjustedCurves” R-package^3^. For inverse-probability for treatment weights “weightit” and “weightitMSM” from the “WeightIt” R-package were used^4^

*Inverse probability of treatment weights*

In the active comparator cohort, a multinomial logistic regression model was used to model the propensity for treatment based on baseline characteristics. In the time-varying cohort, propensity for treatment at each timepoint was modelled based on current and prior covariate status, as well as prior treatment status, in a marginal structural model approach^5^. A logistic regression model was unable to achieve good balance in the time-varying cohort due to the expanding number of covariates. Instead, generalized boosted models were used, which have been shown to outperform logistic regression when there is a large number of covariates and treatment is multicategory^6^. Weights were stabilized, and trimming was done at the 0.1st and 99.9th percentiles to limit the influence of extreme weights.

# Supplementary Table S1: Exposure/treatment definitions

| **Pharmacotherapy** | **Anatomical therapeutic chemical codes** |
| --- | --- |
| Statin | C10AA, C10BX, C10BA01-09, C10BA11-12 |
| Cardioprotective glucose-lowering drugs |  |
| Sodium-glucose cotransporter 2 inhibitor | A10BK, A10BD15-16, A10BD19-21, A10BD23-25, A10BD27, A10BD29 |
| Glucagon-like peptide-1 receptor agonist | A10BJ or A10AE56 |

# Supplementary Table S2: Major adverse cardiovascular event outcome definitions

| **Diagnosis** | **World Health Organization International Classification of Diseases (ICD) codes** |
| --- | --- |
| Myocardial infarction | ICD-8: 410  ICD-10: I21-22 |
| Stroke | ICD-8: 430-31, 433-34, 436  ICD-10: I60-1, I63-4, I690-1, I693-4 |
| Cardiovascular death | ICD-10: I00-99 (registered as primary cause) |

# Supplementary Table S3: Other pharmacotherapy and medical diagnosis definitions

| **Pharmacotherapy** | **Categories/ Anatomical Therapeutic Chemical codes** |
| --- | --- |
| Insulin | A10AB-AE |
| Metformin | A10BA02, A10BD02-03, A10BD05, A10BD07-08, A10BD10-11, A10BD13-18, A10BD20, A10BD22-23, A10BD25-28 |
| Dipeptidyl peptidase-4 inhibitors | A10BH, A10BD07-13, A10BD18-19, A10BD21-22, A10BD24-25, A10BD27-29 |
| Sulfonylureas | A10BB, A10BD01-02, A10BD04, A10BD06 |
| Other glucose-lowering drugs | A10BG, A10BX, A10BF01, A10BD04-06, A10BD09, A10BD12, A10BD14, A10BD17, A10BD26 |
| Other lipid-lowering drugs | C10AB-AD, C10AX, C10BA01-06, C10BA09-12 |
| Beta-blockers | C07, C09BX02, C09BX04-06, C09DX05 |
| Calcium channel blockers | C08, C07FB, C09BB, C09DB, C09BX01, C09BX03-04, C09BX06, C09DX01, C09DX03, C09DX06-08, C09XA53-54, C10BX03, C10BX07, C10BX09, C10BX11, C10BX14, C10BX18, C10BX19 |
| Renin-angiotensin system inhibitors | C09, C10BX04, C10BX06-07, C10BX10-20 |
| Thiazides and thiazide-like drugs | C03A-B, C07B-D, C03EA, C09BA, C09DA, C09BX01, C09BX03, C09BX06, C09DX01, C09DX03, C09DX06-08, C09XA52, C09XA54, C10BX13 |
| Loop diuretics | C03C, C03EB |
| Potassium-sparing diuretics | C03D, C03E |
| Nitrates | C01DA |
| Vitamin K antagonists | B01AA |
| Direct oral anticoagulants | B01AF, B01AE07 |
| Antiplatelets | B01AC, B01AF51, C07FX02-04, C10BX01-02, C10BX04-06, C10BX08, C10BX12 |
| Beta-2 agonist inhalants | R03AC, R03AL, R03AK04-14 |
| Anticholinergic inhalants | R03BB, R03AL |
| Glucocorticoid inhalants | R03BA, R03AK06-14, R03AL08-09, R03AL11-12 |
| Antidepressants | N06A, N06CA |
| Non-steroid anti-inflammatory drugs | M01A, N02AJ08, N02AJ14, N02AJ16, N02AJ19, N02AJ23 |
| Glucocorticoids with systemic action | H02AB |
| **Medical history** | **World Health Organization International Classification of Diseases (ICD) codes/Procedure codes^a^** |
| Nephropathy | ICD8: 24902, 25002  ICD10: E102, E112, E122, E132, E142, N083, N18, N19  NOMESCO codes: KAS, BJFD, BJFZ |
| Neuropathy | ICD8: 24903, 25003, 35401  ICD10: E104, E114, E124, E134, E144, G632, G629,  NOMESCO codes: KQDA, KQDB, KQDG |
| Retinopathy | ICD8: 24901, 25001  ICD10: E103, E113, E123, E133, E143, H360  NOMESCO codes: KCKC10, KCKC15, KCKD05B, KCKD05C |
| Hypertension | ICD-8: 400-404  ICD-10: I10-15 |
| Chronic obstructive pulmonary disease | ICD-8: 49100-04, 49108-09, 49200-01, 49208-09  ICD-10: J41-44 |
| Atrial fibrillation | ICD-8: 42793-94  ICD-10: I48 |
| Venous thromboembolism | ICD-8: 45099, 45100, 45108, 45109, 67101, 67102  ICD-10: I26, I801-03, O223, O871, O882 |
| Heart failure^b^ | ICD-8: 42709, 42710, 42711, 42719 42899, 78249  ICD-10: I110, I130, I132, I420, I426, I427, I428, I429, I50 |
| Aterosclerotic peripheral arterial disease^b^ | ICD-8: 44020-21, 44028-30, 44389, 44399, 44500, 44508-09  ICD-10: I702, I739, L979 |
| Coronary revascularization^b^ |  |
| Percutaneous coronary intervention | Danish classification: 30240, 30350, 30354  NOMESCO codes: KFNF, KFNG |
| Coronary artery bypass graft | Danish classification: 30009, 30019, 30029, 30039, 30049, 30059, 30069, 30079, 30089, 30099, 30109, 30119, 30120, 30129, 30139, 30149, 30159, 30169, 30179, 30189, 30199, 30200  NOMESCO codes: KFNA, KFNB, KFNC, KFND, KFNE |

^a^Procedure codes were defined by Danish surgery and treatment classification codes prior to 1996, where they were replaced by the Nordic Classification of Surgical Procedures (NOMESCO) classification system

^b^Heart failure, peripheral arterial disease, and coronary revascularization definitions were used, alongside the major adverse cardiovascular event subcomponents (Supplementary Table S2), to identify individuals with preexisting cardiovascular disease. Therefore, although specified by ICD-codes here, heart failure, peripheral arterial disease, and coronary revascularization were not used in any of the models for covariate adjustment.

| **Laboratory biomarkers** | **Time in days assessed in relation to index date** | **Codes (the latest value before the index date was used)** |
| --- | --- | --- |
| Hemoglobin A1c, mmol/mol | [-365; 0] | NPU03835, NPU27300 |
| Low-density lipoprotein cholesterol, mmol/L | [-365; 0] | NPU01568, NPU10171, DNK35308 |
| Estimated GFR mL/min per 1.73 m^2^ | [-365; 0] | NPU04998, NPU18016 |

# Supplementary Table S4: Laboratory variables

Estimated glomerular filtration rate (GFR) was calculated from creatinine using the CKD-EPI formula without correction for race.

For women:

Creatinine ≤ 62 μmol/L: eGFR = 144 x (Creatinine/(0∙7 x 88∙4))^-0.329^ x 0∙993^age^

Creatinine > 62 μmol/L: eGFR = 144 x (Creatinine/(0∙7 x 88∙4))^-1.209^ x 0,993^age^

For men

Creatinine ≤ 80 μmol/L: eGFR = 141 x (Creatinine/(0∙9 x 88∙4))^-0.411^ x 0∙993^age^

Creatinine > 80 μmol/L: eGFR = 141 x (Creatinine/(0∙9 x 88∙4))^-1.209^ x 0.993^age^

# Supplementary Table S5: Covariate rationale and collider assessment

***Continues next page***

| **Covariate** | **Rationale** | **Collider risk** |
| --- | --- | --- |
| Sex | Confounder, associated with both prescribing patterns and ASCVD risk | None, fixed characteristic |
| Age | Confounder, strongly associated with both prescribing patterns and ASCVD risk | None, fixed characteristic |
| Descent | Confounder, associated with access to care and ASCVD risk | None, fixed characteristic |
| Year of entry into observation | Confounder, captures secular trends in prescribing and overall ASCVD healthcare following guideline updates | Low, see footnote |
| Income level | Confounder, proxy for health behaviors, adherence, and access to preventive care | Low, see footnote |
| Education level | Confounder, proxy for health behaviors, adherence, and access to preventive care | Low, see footnote |
| Diabetes duration | Confounder, associated with cumulative ASCVD risk and likelihood using cardioprotective drugs | Low, see footnote |
| Hypertension | Confounder, strong independent ASCVD risk factor | Low, see footnote |
| Atrial fibrillation | Confounder, independent risk factor for stroke | Low, see footnote |
| COPD | Confounder, proxy for smoking history and associated systemic inflammation and ASCVD risk | Low, see footnote |
| Venous thromboembolism | Precision variable, retained to reduce residual variance. Associated with coagulation risk and cardiovascular comorbidity. | Low, see footnote |
| Neuropathy | Confounder, marker of diabetes severity and associated ASCVD risk | Low, see footnote |
| Nephropathy | Confounder, marker of diabetes severity and associated ASCVD risk | Low, see footnote |
| Retinopathy | Confounder, marker of diabetes severity and associated ASCVD risk | Low, see footnote |
| Renin-angiotensin system inhibitors | Confounder, proxy for hypertension and diabetic nephropathy. | Low, see footnote |
| Beta-blockers | Confounder, proxy for hypertension and cardiovascular comorbidity | Low, see footnote |
| Calcium channel blockers | Confounder, proxy for hypertension | Low, see footnote |
| Thiazides / thiazide-like drugs | Confounder, proxy for hypertension | Low, see footnote |
| Loop diuretics | Confounder, proxy for advanced cardiovascular or renal disease | Low, see footnote |
| Potassium-sparing diuretics | Confounder, proxy for heart failure | Low, see footnote |
| Nitrates | Confounder, proxy for advanced cardiovascular disease | Low, see footnote |
| Antiplatelets | Confounder, independent treatment in secondary prevention. Proxy for ASCVD risk and management intensity. | Low, see footnote |
| Vitamin K antagonists | Confounder, proxy for atrial fibrillation and thromboembolic burden | Low, see footnote |
| Direct oral anticoagulants | Confounder, proxy for atrial fibrillation and thromboembolic burden | Low, see footnote |
| Other lipid-lowering drugs | Confounder, proxy for high ASCVD risk and lipid management intensity | Low, see footnote |
| Beta-2 agonist inhalants | Confounder, proxy for smoking history, COPD/asthma, and associated ASCVD risk | Low, see footnote |
| Anticholinergic inhalants | Confounder, proxy for smoking history, COPD/asthma, and associated ASCVD risk | Low, see footnote |
| Glucocorticoid inhalants | Confounder, proxy for smoking history, COPD/asthma, and associated ASCVD risk | Low, see footnote |
| Antidepressants | Confounder, proxy for depression and associated ASCVD risk | Low, see footnote |
| Non-steroid antiinflammatory drugs | Confounder, proxy for inflammatory conditions and associated ASCVD risk | Low, see footnote |
| Systemic glucocorticoids | Confounder, proxy for inflammatory disease and associated metabolic and ASCVD risk | Low, see footnote |
| Hemoglobin A1c | Confounder, marker of glycaemic control and diabetes severity associated with ASCVD risk. Excluded from main models due to missing values; included in sensitivity analyses. | Low, see footnote |
| Estimated glomerular filtration rate | Confounder, marker of renal function associated with ASCVD risk and prescribing patterns. Excluded from main models due to missing values; included in sensitivity analyses. | Low, see footnote |
| Low-density lipoprotein cholesterol | Mediator, low-density lipoprotein cholesterol reduction is the primary mechanism by which statins reduce ASCVD. Adjustment could inappropriately attenuate effect estimates in statin users. Excluded from models. Included for adjustment in one sensitivity analysis. | Mediator |

***Supplementary Table S5 continued***

In the active comparator cohort variables were ascertained prior to the index date (initiation of cardioprotective glucose-lowering drug or dipeptidyl peptidase-4 inhibitor) and are therefore not caused by the index-date treatment assignment. For prevalent statin users, some comorbidity diagnoses and concomitant medications may postdate statin initiation; however, none of those included in the main model were assessed to be plausibly caused by statin use and are thus unlikely to meaningfully bias estimates. Collider bias was therefore considered low for all covariates included in the main model. Abbreviations: ASCVD: atherosclerotic cardiovascular disease; COPD: chronic obstructive pulmonary disease.

# Supplementary Table S6: Sizes of treatment groups at each timepoint in the time-varying cohort

| **Timepoint** | **Treatment groups** | | | |
| --- | --- | --- | --- | --- |
|  | **No cardioprotective drug** | **Cardioprotective glucose-lowering drug** | **Statin** | **Cardioprotective glucose-lowering drug and statin** |
| Timepoint 0 – index date/entry into observation | 13,268 | 886 | 57,808 | 4,891 |
| Timepoint 1 – one year after index date | 11,155 | 774 | 47,525 | 4,838 |
| Timepoint 2 – two years after index date | 9,282 | 688 | 39,915 | 4,741 |
| Timepoint 3 – three years after index date | 7,788 | 698 | 33,273 | 4,944 |
| Timepoint 4 – four years after index date | 6,440 | 711 | 27,474 | 5,144 |
| Timepoint 5 – five years after index date | 5,295 | 687 | 22,188 | 5,330 |
| Timepoint 6 – six years after index date | 4,269 | 680 | 17,655 | 5,687 |
| Timepoint 7 – seven years after index date | 3,375 | 730 | 13,720 | 5,998 |
| Timepoint 8 – eight years after index date | 2,618 | 740 | 10,442 | 6,159 |

Number of individuals in each treatment category at baseline and at each of the eight yearly status updates in the time varying cohort.

# Supplementary Table S7: Stepwise adjusted models for the primary outcome

| **Active comparator cohort** | | | | |
| --- | --- | --- | --- | --- |
| **Treatment**  **(reference is dipeptidyl peptidase-4 inhibitor)** | **Model 1**  **unadjusted** | **Model 2**  **adjusted for heritable variables** | **Model 3**  **adjusted for general demographic variables** | **Model 4**  **fully adjusted, including medical history and pharmacotherapies** |
| Cardioprotective glucose-lowering drug | 0.57 (0.47-0.70) | 0.74 (0.61-0.91) | 0.81 (0.65-1.00) | 0.82 (0.67-1.02) |
| Statin | 0.82 (0.72-0.93) | 0.81 (0.71-0.92) | 0.82 (0.72-0.94) | 0.85 (0.74-0.97) |
| Cardioprotective glucose-lowering drug and statin | 0.53 (0.45-0.61) | 0.62 (0.54-0.72) | 0.69 (0.59-0.81) | 0.71 (0.60-0.84) |
| **Time-varying cohort** | | | | |
| **Treatment**  **(reference is no cardioprotective drug)** | **Model 1**  **unadjusted** | **Model 2**  **adjusted for heritable variables** | **Model 3**  **adjusted for general demographic variables** | **Model 4**  **fully adjusted, including medical history and pharmacotherapies** |
| Cardioprotective glucose-lowering drug | 0.57 (0.51-0.64) | 0.78 (0.70-0.87) | 0.76 (0.68-0.85) | 0.77 (0.69-0.86) |
| Statin | 0.64 (0.62-0.66) | 0.71 (0.68-0.73) | 0.70 (0.68-0.72) | 0.73 (0.70-0.75) |
| Cardioprotective glucose-lowering drug and statin | 0.43 (0.41-0.45) | 0.59 (0.55-0.62) | 0.55 (0.52-0.58) | 0.57 (0.54-0.60) |

Cox proportional hazard ratios with 95% confidence intervals for the primary outcome of 3-point major adverse cardiovascular events (MACE) in models that incrementally include more covariates. Model 1 is unadjusted. Model 2 includes heritable characteristics: sex, age, and descent. Model 3 additionally includes other general demographic characteristics: education level, income level, time since first glucose-lowering drug, and year of entry into observation. Model 4, as reported in the main article, is fully adjusted using all covariates in the baseline tables. In contrast to Model 3, Model 4 also includes medical history and concomitant pharmacotherapy characteristics in adjustments. No cardioprotective drug denotes no use of either cardioprotective glucose-lowering drug or statin.

# Supplementary Figure S1: Flowchart for the active comparator cohort

***Inclusion criteria:*** Individuals who dispensed a prescription for metformin as their first glucose-lowering drug class and were alive within the observation period: December 10, 2012 through December 31, 2021.

Excluded

 	Never filled a prescription for any second glucose-lowering drug class

n=147,341

 	Second glucose-lowering class dispensed prior to December 10, 2012

n=55,250

 	Second glucose-lowering class dispensed in Danish pharmacy after being registered as emigrated

n=74

 	ICD code for type 1 diabetes

n=2,373

 	ICD code gestational diabetes

n=383

 	Insulin dispensed prior to age 30

n=381

 	Any glucose-lowering drug dispensed prior to age 15

n=17

 	More than one class of a new glucose-lowering drug class dispensed on the same date

n=355

 	Second class was not dipeptidyl peptidase-4 inhibitor, sodium glucose cotransporter 2 inhibitor, or a glucagon-like peptide-1 receptor agonist

n=19,476

Individuals identified by inclusion criteria

n=300,935

Cardioprotective glucose-lowering and statin treatment

n=6,633

Cardioprotective glucose-lowering drug

Dipeptidyl peptidase-4 inhibitor

Individuals with type 2 diabetes as per above criteria

n=75,285

Excluded

 	No history of cardiovascular disease before index date

n=59,795

Individuals with type 2 diabetes and established cardiovascular disease

n=15,490

Excluded

 	<2 years of lookback at index date n=86

***Index date*** was the date when a second glucose-lowering class was first dispensed within the observation period.

Distribution of treatment at index date

Dipeptidyl peptidase-4 inhibitor and statin

**Abbreviations:** ICD: World Health Organization International Classification of Diseases

n=5,362

n=1,868

n=1,541

# Supplementary Figure S2: Flowchart for the time-varying cohort

Individuals identified by inclusion criteria

n=437,706

Statin

n=57,808

Cardioprotective glucose-lowering drug

No cardioprotective glucose-lowering drug or statin

Cardioprotective glucose-lowering drug and statin

n=4,891

***Inclusion criteria:*** Living individuals who dispensed a prescription for any glucose lowering drug within four years prior to January 1, 2013, or at any time within the observation period: January 1, 2013 through December 31, 2021.

Excluded

 	ICD code for type 1 diabetes

n=31,643

 	ICD code for gestational diabetes

n=1,593

 	Insulin dispensed prior to age 30

n=7,040

 	Any glucose-lowering drug dispensed prior to age 15

n=182

 	First glucose-lowering drug dispensed after the last timepoint, January 1, 2020

n=38,252

 	Individuals who died or emigrated within the calendar year of their first dispensed glucose-lowering drug. Thus, they were censored prior to January 1^st^ in the succeeding calendar year, which would have become Timepoint 0

n=4,017



Individuals with type 2 diabetes as per above criteria

n=354,979

Excluded

 	No history of cardiovascular disease before index date

n=277,734

<2 years of lookback at index date

n=392

Individuals with type 2 diabetes and established cardiovascular disease

n=76,853

***Index date*** was January 1, 2013, for those who dispensed a prescription for a glucose lowering drug prior to that date. Otherwise, if the first prescription was within the observation period, the index date was on January 1^st^ in the succeeding calendar year.

Distribution of treatment at first timepoint/index date (Timepoint 0)

n=13,268

# Supplementary Figure S3: Covariate balances at baseline before and after weighting in the active comparator cohort

n=886

**Abbreviations:** ICD: World Health Organization International Classification of Diseases

Loveplot with covariates listed in order of highest to lowest standardized mean difference after weighting (blue points) with red points indicating balances before weighting. The dotted line indicates a threshold of 0.1.

*Categorical or binary variable.

# Supplementary Figure S4: Covariate balances at before and after weighting at the first timepoint (V0) in the time-varying cohort

Loveplot with covariates listed in order of highest to lowest standardized mean difference after weighting (blue points) with red points indicating balances before weighting. The dotted line indicates a threshold of 0.1.

*Categorical or binary variable.

# Supplementary Figure S5: Covariate balances at before and after weighting at the second timepoint (V1) after one year in the time-varying cohort

Loveplot with covariates listed in order of highest to lowest standardized mean difference after weighting (blue points) with red points indicating balances before weighting. The dotted line indicates a threshold of 0.1.*Categorical or binary variable.

# Supplementary Figure S6: Covariate balances at before and after weighting at the third timepoint (V2) after two years in the time-varying cohort

***Continues next page***

***Supplementary Figure S6 continued***

Loveplot with covariates listed in order of highest to lowest standardized mean difference after weighting (blue points) with red points indicating balances before weighting. The dotted line indicates a threshold of 0.1.*Categorical or binary variable.

# Supplementary Figure S7: Covariate balances at before and after weighting at the fourth timepoint (V3) after three years in the time-varying cohort

***Continues next page***

***Supplementary Figure S7 continued***

Loveplot with covariates listed in order of highest to lowest standardized mean difference after weighting (blue points) with red points indicating balances before weighting. The dotted line indicates a threshold of 0.1.*Categorical or binary variable.

# Supplementary Figure S8: Covariate balances at before and after weighting at the fifth timepoint (V4) after four years in the time-varying cohort

***Continues next page***

***Supplementary Figure S8 continues***

Loveplot with covariates listed in order of highest to lowest standardized mean difference after weighting (blue points) with red points indicating balances before weighting. The dotted line indicates a threshold of 0.1. *Categorical or binary variable.

# Supplementary Figure S9: Covariate balances at before and after weighting at the sixth timepoint (V5) after five years in the time-varying cohort

***Continues next page***

***Supplementary Figure S9 continued***

Loveplot with covariates listed in order of highest to lowest standardized mean difference after weighting (blue points) with red points indicating balances before weighting. The dotted line indicates a threshold of 0.1.*Categorical or binary variable.

# Supplementary Figure S10: Covariate balances at before and after weighting at the seventh timepoint (V6) after six years in the time-varying cohort

***Continues next page***

***Continues next page***

***Supplementary Figure S10 continued***

***Supplementary Figure S10 continued***

Loveplot with covariates listed in order of highest to lowest standardized mean difference after weighting (blue points) with red points indicating balances before weighting. The dotted line indicates a threshold of 0.1.*Categorical or binary variable.

# Supplementary Figure S11: Covariate balances at before and after weighting at the eighth timepoint (V7) after seven years in the time-varying cohort

***Continues next page***

***Continues next page***

***Supplementary Figure S11 continued***

***Supplementary Figure S11 continued***

Loveplot with covariates listed in order of highest to lowest standardized mean difference after weighting (blue points) with red points indicating balances before weighting. The dotted line indicates a threshold of 0.1.*Categorical or binary variable.

# Supplementary Figure S12: Covariate balances at before and after weighting at the ninth timepoint (V8) after eight years in the time-varying cohort

***Continues next page***

***Supplementary Figure S12 continued***

***Continues next page***

***Supplementary Figure S12 continued***

Loveplot with covariates listed in order of highest to lowest standardized mean difference after weighting (blue points) with red points indicating balances before weighting. The dotted line indicates a threshold of 0.1. *Categorical or binary variable.

# Supplementary Figure S13: Weight adjusted model in the time-varying cohort with unbalanced covariates added to the outcome model

Hazard ratios for secondary MACE adjusted additionally for age, history of hypertension and nephropathy, and use of insulin and loop diuretics after covariates were balanced as seen in Supplementary Figures S4-S12 for the time-varying cohort. Covariates that at any timepoint had standardized mean differences >0.1 were included for adjustments. Inverse probability for treatment weighting was used to balance covariates. Cardioprotective glucose-lowering drug is either sodium-glucose cotransporter-2 inhibitor or glucagon-like peptide-1 receptor agonist. No cardioprotective drug denotes no use of statin or cardioprotective glucose-lowering drug. Abbreviations: MACE: Major adverse cardiovascular events; HR: Hazard ratio; CI: Confidence interval; DPP4i: Dipeptidyl peptidase-4 inhibitor; CPGLD: Cardioprotective glucose-lowering drug.

# Supplementary Figure S14: Cumulative incidence of major adverse cardiovascular events with use of cardioprotective glucose-lowering drug and statin with confidence intervals

Cause-specific cumulative incidence of secondary MACE for the active comparator cohort adjusted for covariates listed in the baseline characteristics table by standardization through the G-formula. Death due to noncardiovascular causes was treated as competing risk. Cardioprotective glucose-lowering drug is either sodium-glucose cotransporter-2 inhibitor or glucagon-like peptide-1 receptor agonist. Smaller transparent lines indicate corresponding 95% confidence intervals. Abbreviations: MACE: Major adverse cardiovascular events; DPP4i: Dipeptidyl peptidase-4 inhibitor; CPGLD: Cardioprotective glucose-lowering drug; CI: Confidence interval.

# Supplementary Figure S15: Association of cardioprotective glucose-lowering drug and statin with major adverse cardiovascular events and all-cause mortality by year of entry in the time-varying cohort

Cox proportional hazards model estimates for secondary MACE and all-cause mortality adjusted for covariates in the baseline characteristics table for the time-varying cohort, when stratified by year of entry into observation (2019–2021, 2016–2018, and 2012–2015). Cardioprotective glucose-lowering drug is either sodium-glucose cotransporter-2 inhibitor or glucagon-like peptide-1 receptor agonist. No cardioprotective drug denotes no use of statin or cardioprotective glucose-lowering drug. Abbreviations: MACE: Major adverse cardiovascular events; HR: Hazard ratio; CI: Confidence interval; DPP4i: Dipeptidyl peptidase-4 inhibitor; CPGLD: Cardioprotective glucose-lowering drug.

# Supplementary Figure S16: Association of cardioprotective glucose-lowering drug and statin with major adverse cardiovascular events and all-cause mortality additionally adjusted for laboratory values

Cox proportional hazards model estimates for secondary MACE and all-cause mortality adjusted for covariates in the baseline characteristics tables for the active comparator and the time-varying cohorts, with hemoglobin A1c and eGFR added to adjustments (upper section of upper and lower panels) and with LDL cholesterol also added to adjustments (lower section of upper and lower panels). Cardioprotective glucose-lowering drug is either sodium-glucose cotransporter-2 inhibitor or glucagon-like peptide-1 receptor agonist. No cardioprotective drug denotes no use of statin or cardioprotective glucose-lowering drug. Abbreviations: MACE: Major adverse cardiovascular events; HR: Hazard ratio; CI: Confidence interval; DPP4i: Dipeptidyl peptidase-4 inhibitor; CPGLD: Cardioprotective glucose-lowering drug; eGFR: estimated glomerular filtration rate; LDL: low-density lipoprotein.

# Supplementary Figure S17: Association of cardioprotective glucose-lowering drug and statin with major adverse cardiovascular events and all-cause mortality by duration of statin treatment in the active comparator cohort

Cox proportional hazards model estimates for secondary MACE and all-cause mortality adjusted for covariates in the baseline characteristics table when stratified by duration of statin treatment at index date (<1 year, 1-3 years, 3-5 years, >5 years) for the active comparator cohort. Cardioprotective glucose-lowering drug is either sodium-glucose cotransporter-2 inhibitor or glucagon-like peptide-1 receptor agonist. No cardioprotective drug denotes no use of statin or cardioprotective glucose-lowering drug. Abbreviations: MACE: Major adverse cardiovascular events; HR: Hazard ratio; CI: Confidence interval; DPP4i: Dipeptidyl peptidase-4 inhibitor; CPGLD: Cardioprotective glucose-lowering drug.

# Supplementary Figure S18: Association of cardioprotective glucose-lowering drug and statin with major adverse cardiovascular events and all-cause mortality by duration of statin treatment in the time-varying cohort

Cox proportional hazards model estimates for secondary MACE and all-cause mortality adjusted for covariates in the baseline characteristics table when stratified by duration of statin treatment at index date (<1 year, 1-3 years, 3-5 years, >5 years) for the time-varying cohort. Cardioprotective glucose-lowering drug is either sodium-glucose cotransporter-2 inhibitor or glucagon-like peptide-1 receptor agonist. No cardioprotective drug denotes no use of statin or cardioprotective glucose-lowering drug. Abbreviations: MACE: Major adverse cardiovascular events; HR: Hazard ratio; CI: Confidence interval; DPP4i: Dipeptidyl peptidase-4 inhibitor; CPGLD: Cardioprotective glucose-lowering drug.

# Supplementary Figure S19: Statin therapy defined as >80% proportion days covered in the preceding year for the time-varying cohort

Cox proportional hazards model estimates for secondary MACE and all-cause mortality adjusted for covariates in the baseline characteristics table for the time-varying cohort with statin use classified as >80% proportion days covered in the preceding year. Cardioprotective glucose-lowering drug is either sodium-glucose cotransporter-2 inhibitor or glucagon-like peptide-1 receptor agonist. No cardioprotective drug denotes no use of statin or cardioprotective glucose-lowering drug. Abbreviations: MACE: Major adverse cardiovascular events; HR: Hazard ratio; CI: Confidence interval; DPP4i: Dipeptidyl peptidase-4 inhibitor; CPGLD: Cardioprotective glucose-lowering drug.

# Supplementary Figure S20: Association of cardioprotective glucose-lowering drug and statin with major adverse cardiovascular events and all-cause mortality in individuals with antiplatelets and renin-angiotensin system inhibitors at baseline

Cox proportional hazards model estimates for secondary MACE and all-cause mortality adjusted for covariates in the baseline characteristics tables for the active comparator and the time-varying cohorts in individuals treated with antiplatelets and renin-angiotensin system inhibitors at baseline. Cardioprotective glucose-lowering drug is either sodium-glucose cotransporter-2 inhibitor or glucagon-like peptide-1 receptor agonist. No cardioprotective drug denotes no use of statin or cardioprotective glucose-lowering drug. Abbreviations: MACE: Major adverse cardiovascular events; HR: Hazard ratio; CI: Confidence interval; DPP4i: Dipeptidyl peptidase-4 inhibitor; CPGLD: Cardioprotective glucose-lowering drug.

# Supplementary Figure S21: Association of cardioprotective glucose-lowering drug and statin with major adverse cardiovascular events and all-cause mortality using a 90-day grace period

Cox proportional hazards model estimates for secondary MACE adjusted for covariates in the baseline characteristics tables for the active comparator and the time-varying cohorts with a 90-day grace period used to classify new myocardial infarction or stroke instead of 30 days. Cardioprotective glucose-lowering drug is either sodium-glucose cotransporter-2 inhibitor or glucagon-like peptide-1 receptor agonist. No cardioprotective drug denotes no use of statin or cardioprotective glucose-lowering drug. Abbreviations: MACE: Major adverse cardiovascular events; HR: Hazard ratio; CI: Confidence interval; DPP4i: Dipeptidyl peptidase-4 inhibitor; CPGLD: Cardioprotective glucose-lowering drug.

# Supplementary Figure S22: Association of cardioprotective glucose-lowering drug and statin with nonfatal myocardial infarction, stroke, and cardiovascular death in individuals with prior myocardial infarction

Cox proportional hazards model estimates for nonfatal myocardial infarction, stroke, and cardiovascular death adjusted for covariates in the baseline characteristics tables for the active comparator (left column) and the time-varying (right column) cohorts. The cohort of individuals was restricted to those with prior myocardial infarction. Cardioprotective glucose-lowering drug is either sodium-glucose cotransporter-2 inhibitor or glucagon-like peptide-1 receptor agonist. No cardioprotective drug denotes no use of statin or cardioprotective glucose-lowering drug. Abbreviations: HR: Hazard ratio; CI: Confidence interval; DPP4i: Dipeptidyl peptidase-4 inhibitor; CPGLD: Cardioprotective glucose-lowering drug.

# Supplementary Figure S23: Association of cardioprotective glucose-lowering drugs and statin with major adverse cardiovascular events using Fine and Gray models

Subdistribution hazard ratios for secondary MACE in the active comparator cohort from Fine and Gray models for sodium-glucose cotransporter-2 inhibitor (upper panel), glucagon-like peptide-1 receptor agonist (middle panel), and both pooled as cardioprotective glucose-lowering drug (lower panel) in combination with statins. Noncardiovascular mortality was treated as competing risk. The models were adjusted for covariates in the baseline characteristics table for the active comparator cohort. Abbreviations: SGLT2i: sodium-glucose cotransporter-2 inhibitor; GLP1-RA: glucagon-like peptide-1 receptor agonist; DPP4i: Dipeptidyl peptidase-4 inhibitor; CPGLD: Cardioprotective glucose-lowering drug. MACE: Major adverse cardiovascular events; sdHR: Subdistribution hazard ratio; CI: Confidence interval.

# Supplementary Figure S24: Association of cardioprotective glucose-lowering drugs and statin with major adverse cardiovascular events by year of entry using Fine and Gray models

Subdistribution hazard ratios for secondary MACE in the active comparator cohort from Fine and Gray models stratified by year of entry into observation (2019–2021, 2016–2018, and 2012–2015). Noncardiovascular mortality was treated as competing risk. The models were adjusted for covariates in the baseline characteristics table for the active comparator cohort. Cardioprotective glucose-lowering drug is either sodium-glucose cotransporter-2 inhibitor or glucagon-like peptide-1 receptor agonist. Abbreviations: DPP4i: Dipeptidyl peptidase-4 inhibitor; CPGLD: Cardioprotective glucose-lowering drug. MACE: Major adverse cardiovascular events; sdHR: Subdistribution hazard ratio; CI: Confidence interval.

# Supplementary Figure S25: Association of cardioprotective glucose-lowering drugs and statin with major adverse cardiovascular events by sex using Fine and Gray models

Subdistribution hazard ratios for secondary MACE in the active comparator cohort from Fine and Gray models stratified by sex. Noncardiovascular mortality was treated as competing risk. The models were adjusted for covariates in the baseline characteristics table for the active comparator cohort. Cardioprotective glucose-lowering drug is either sodium-glucose cotransporter-2 inhibitor or glucagon-like peptide-1 receptor agonist. Abbreviations: DPP4i: Dipeptidyl peptidase-4 inhibitor; CPGLD: Cardioprotective glucose-lowering drug. MACE: Major adverse cardiovascular events; sdHR: Subdistribution hazard ratio; CI: Confidence interval.

# References

1.	Therneau T. A Package for Survival Analysis in R. R package version 3.5-7. Published online 2023. <https://CRAN.R-project.org/package=survival>.

2.	Therneau T, Crowson C, Atkinson E. Using Time Dependent Covariates and Time Dependent Coefficients in the Cox Model. Accessed March 11, 2025. https://cran.r-project.org/web/packages/survival/vignettes/timedep.pdf

3.	Denz R, Klaaßen‐Mielke R, Timmesfeld N. A comparison of different methods to adjust survival curves for confounders. *Stat Med*. 2023;42(10):1461-1479. doi:10.1002/sim.9681

4.	Greifer N. WeightIt: Weighting for Covariate Balance in Observational Studies. R package version 0.14.2. Published online 2023. https://CRAN.R-project.org/package=WeightIt

5.	Hernán MA, Brumback B, Robins JM. Marginal Structural Models to Estimate the Joint Causal Effect of Nonrandomized Treatments. *J Am Stat Assoc*. 2001;96(454):440-448. doi:10.1198/016214501753168154

6.	McCaffrey DF, Griffin BA, Almirall D, Slaughter ME, Ramchand R, Burgette LF. A tutorial on propensity score estimation for multiple treatments using generalized boosted models. *Stat Med*. 2013;32(19):3388-3414. doi:10.1002/sim.5753
